# Transparent Ceramic@Sapphire Composites for High‐Power Laser‐Driven Lighting

**DOI:** 10.1002/advs.202505232

**Published:** 2025-04-29

**Authors:** Guoyu Xi, Shisheng Lin, Tongjie Shen, Tao Pang, Zikang Yu, Yang Peng, Lingwei Zeng, Yuxiang Ke, Zhehong Zhou, Ronghua Chen, Feng Huang, Daqin Chen

**Affiliations:** ^1^ College of Physics and Energy Fujian Normal University Fuzhou Fujian 350117 P. R. China; ^2^ Huzhou Key Laboratory of Materials for Energy Conversion and Storage College of Science Huzhou University Huzhou Zhejiang 313000 P. R. China; ^3^ School of Mechanical Science and Engineering Huazhong University of Science and Technology Wuhan Hubei 430074 P. R. China; ^4^ School of Chemistry and Chemical Engineering Hunan University of Science and Technology Xiangtan Hunan 411201 P. R. China; ^5^ Fujian Provincial Collaborative Innovation Center for Advanced High‐Field Superconducting Materials and Engineering Fujian Normal University Fuzhou Fujian 350117 P. R. China; ^6^ Fujian Provincial Engineering Technology Research Center of Solar Energy Conversion and Energy Storage Fujian Normal University Fuzhou Fujian 350117 P. R. China

**Keywords:** laser‐driven lighting, luminescent materials, transparent ceramics

## Abstract

Although phosphor ceramics are promising candidates for high‐power laser lighting applications, their performance is seriously restricted by luminance saturation effects. This study proposes a novel transparent ceramic@sapphire composite material design, fabricated via a straightforward high‐temperature sintering process, which differs from the conventional approach of incorporating high‐thermal‐conductivity microcrystalline grains. This kind of composite can effectively avoid luminescence grain dilution, and deliver significantly enhanced thermal conductivity (36.9 W·m^−1^K^−1^) alongside superior luminescence performance. This strategy demonstrates exceptional versatility across various ceramic systems, delivering luminescence improvements of 152–319% and enhancing luminance saturation thresholds by 100–233%, relative to traditional ceramic converters. Using Lu_2‐x_CaMg_2_Si_3_O_12_: xCe^3+^@sapphire as a representative example, the optimized composite enables substantial enhancements in luminous flux (5902 lm) and luminous efficacy (148 lm W^−1^) under blue laser excitation. Compared with commercial counterparts, practical applications in automotive headlights further validate the potential of this design, offering far higher luminance intensity, extended illumination distances (> 400 m), and more uniform color distribution. This study provides a scalable and universal strategy for advancing next‐generation solid‐state lighting.

## Graphical abstract

This work introduces an innovative strategy for high‐power laser lighting by integrating a phosphor ceramic layer with a high‐thermal‐conductivity sapphire substrate through a simple high‐temperature sintering process. This approach enhances luminous flux (152–319%) and increases the luminescence saturation threshold (100–233%), making it versatile across various high‐performance lighting systems.

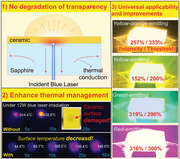

## 1 Introduction

With the global objective of achieving net‐zero emissions by 2050, ≈29% of nations over the world have adopted energy‐efficient lighting technologies. Among emerging advancements, the “blue laser diode (LD) + color converter” technology has gained significant attention as a next‐generation high‐power solid‐state lighting solution, due to its superior energy density output, stable wall‐plug efficiency, exceptional brightness, and precise directional emission.^[^ ^1^, ^2^, ^3^, ^4^, ^5^, ^6^, ^7^ ^]^ Within this framework, phosphor ceramics stand out among various all‐inorganic photonic converters as highly suitable for high‐power laser‐driven lighting applications, owing to their superior laser irradiation resistance, excellent physical/chemical stability, and controllable microstructures.^[^ ^8^, ^9^, ^10^, ^11^ ^]^ However, a critical challenge in the development of phosphor ceramics for laser‐driven lighting, as with other photonic converters, lies in restraining the “luminance saturation” effect.^[^ ^12^, ^13^, ^14^, ^15^, ^16^, ^17^ ^]^

Luminance saturation, primarily caused by thermal quenching (thermal saturation) and optical excitation intensity quenching (optical saturation), necessitates effective thermal management strategies, which helps to overcome this limitation in laser‐driven phosphor ceramics.^[^ ^18^, ^19^, ^20^ ^]^ Although incorporating high‐thermal‐conductivity crystalline grains (e.g., AlN, BN, Al_2_O_3_, MgO) into the ceramic bulk serves as a common approach, it still encounters several inherent challenges.^[^ ^1^ ^]^ Degradation of transparency^[^ ^21^, ^22^ ^]^: The introduction of high‐thermal‐conductivity secondary phases, typically in the form of microcrystals, significantly reduces the visible light transparency of phosphor ceramics, particularly undermining their performance in transmission‐mode laser‐driven lighting. For example, introducing 37.5 wt.% Al_2_O_3_ into Y_3_Al_5_O_12_: Ce^3+^ ceramics reduces visible light transmittance from 90% to 65%.^[^ ^2^, ^19^ ^]^ Dilution of luminescent grains: The number of luminescent grains per unit excitation area decreases with the increased content of the secondary phase. When the content of the secondary phase exceeds a certain threshold, the emission efficiency decreases significantly, thus restricting the permissible added content of high‐thermal‐conductivity phases.^[^ ^3^, ^19^, ^23^, ^24^, ^25^, ^26^ ^]^ Localized agglomeration of raw materials: The addition of secondary‐phase materials normally results in localized agglomeration, significantly increasing experimental complexity and experimental duration.^[^ ^27^, ^28^ ^]^ Similar challenges have also been observed in phosphor‐in‐glass (PiG) film systems. Though incorporation such as Al_2_O_3_ and BN improves luminance saturation threshold, excessive loading leads to trade‐offs between transparency and luminous efficacy, which aligns with the above limitations of phosphor ceramics.^[^ ^29^, ^30^ ^]^ As such, there has been an urgent demand for a generalized material design strategy that not only mitigates the luminance saturation effect but also enhances the efficiency of manufacturing functional photonic conversion composites.

Here, we present an innovative transparent ceramic@sapphire composite material architecture to tackle the problem mentioned above. Based on a facile high‐temperature sintering process, this design integrates a phosphor ceramic layer with a sapphire substrate, combining excellent luminescent properties with superior thermal dissipation. Compatible with green‐, yellow‐, yellow‐orange‐, and red‐emitting phosphor systems, it delivers universal improvements (152–319%) in performance. Taking Lu_2‐x_CaMg_2_Si_3_O_12_: xCe^3+^ (abbreviated as LCMS: Ce) @ sapphire as an example, this composite achieves luminous flux (LF) of 3778 lm under 40 W blue laser excitation, a 257% enhancement compared to LCMS: Ce ceramics, and retains 75% of its luminescent intensity at 200 °C. The optimized LCMS: Ce @ sapphire, featuring a 0.4 mm ceramic layer, further enhances performance with LF of 5902 lm, luminous efficacy (LE) of 148 lm W^−1^, chromaticity coordinates of (0.4101, 0.3944), and a correlated color temperature (CCT) of 3427 K. By overcoming the inherent limitations of traditional ceramic converters, the composite material design provides a versatile and scalable solution for next‐generation high‐power lighting applications, such as automotive laser headlights, where stable, efficient, and long‐range illumination is paramount.^[^ ^31^, ^32^ ^]^

## 2 Results

**Figure 1:**
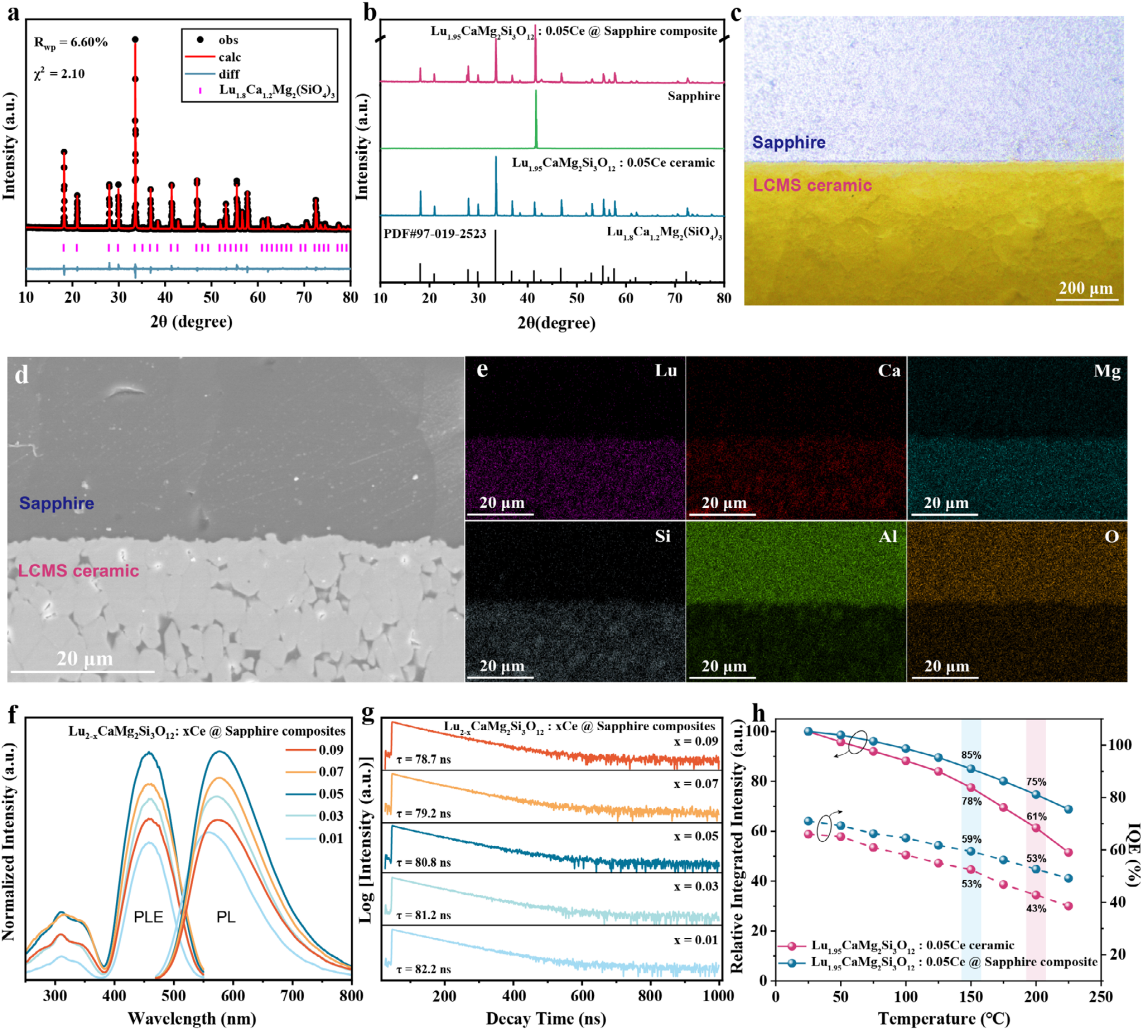
Phase identification, microstructure, and luminescence properties of LCMS: Ce @ Sapphire composite. a) Rietveld refinement analysis of LCMS: 0.05Ce ceramic layer. b) XRD patterns of LCMS: Ce @ Sapphire composite, Sapphire, and LCMS: 0.05Ce ceramic. c) Fluorescence microscopy of LCMS: Ce @ Sapphire composite. d) Cross‐sectional SEM observations and e) EDS surface mapping revealing the elemental distribution of Lu, Ca, Mg, Si, Al, and O for representative LCMS: Ce @ Sapphire composite. Ce^3+^ concentration‐dependent f) PL/PLE spectra and g) decay curves of LCMS: Ce @ Sapphire composite. h) Tempature‐dependent relative integrated intensity and internal quantum efficiency (IQE) of LCMS: 0.05Ce ceramic and LCMS: 0.05Ce @ Sapphire composite.

A series of ceramic@sapphire composites were synthesized based on the simple high‐temperature sintering. Among them, high‐purity transparent LCMS: 0.05Ce ceramic has been successfully fabricated for the first time (Figure S1 and Note S1, Supporting Information). The X‐ray diffraction (XRD) pattern and corresponding Rietveld refinement analysis confirm that the diffraction peaks of typical LCMS: Ce ceramic layer align with cubic Lu_1.8_Ca_1.2_Mg_2_Si_3_O_12_ (PDF 97‐019‐2523), belonging to the space group I$\bar{a}$3d (**Figure** **1**a; Figure S2, Supporting Information). For instance, the refined fitting parameters of the LCMS: 0.05Ce ceramic layer are R_wp_ = 6.60%, χ^2^ = 2.10, with a unit cell parameter a = 11.947 Å, slightly smaller than that of the Lu_1.8_Ca_1.2_Mg_2_Si_3_O_12_ phase (a = 11.976 Å). This reduction is attributed to the smaller ionic radius of Lu^3+^ compared to Ca^2+^, combined with the small content of Ce^3+^ substituting for Lu^3+^ (rLu^3+^ = 0.977 Å, rCa^2+^ = 1.120 Å, rCe^3+^ = 1.143 Å).^[^ ^33^ ^]^ Correspondingly, the LCMS: Ce @ Sapphire composite shows no evidence of impurity phases under the proposed material architecture design (Figure 1b). Moreover, fluorescence microscopy reveals a distinct boundary with well‐isolated phase layers (Figure 1c). Clearly illustrated from cross‐sectional SEM observations, the grains in the LCMS: Ce ceramic layer are tightly bonded to the sapphire substrate, rendering the ceramic layer resistant to being scraped off by nails and facilitating effective heat transfer horizontally/vertically from the phosphor ceramic layer to the surrounding air (Figure 1d; Figure S3, Supporting Information). Further, energy‐dispersive spectroscopy (EDS) surface mapping confirms the respective chemical compositions, indicating no obvious mutual corrosion during the co‐sintering process (Figure 1e).

The spectroscopic properties of the LCMS: Ce @ Sapphire composites were also analyzed. As shown in Figure 1f, the photoluminescence (PL) and photoluminescence excitation (PLE) spectra, dependent on Ce^3+^ concentration, exhibit the typical Ce^3+^ dipole‐allowed 4f↔5d transitions^[^ ^34^, ^35^, ^36^ ^]^ (Figure S4, Supporting Information). The LCMS:0.05Ce @ sapphire achieves the highest yellow‐orange emission intensity centered at 580 nm. An overlap between the PLE and PL spectra results in an decreased emission at shorter wavelengths, ultimately causing a slight redshift in the spectrum with the Ce^3+^ concentration increasing from x = 0.01 to x = 0.09 (Figure S5, Supporting Information). In Figure 1g, the luminescence decay curves of the LCMS: Ce @ Sapphire composites show that the average decay times decrease with the increased Ce^3+^ content, primarily due to energy transfer between Ce^3+^ ions.^[^ ^37^ ^]^ The thermal conductivities of the LCMS: Ce ceramic and LCMS: Ce @ sapphire composites (thickness: 2.0 mm) are 2.3 W (m K)^−1^ and 36.9 W (m K)^−1^, respectively (Figure S6, Supporting Information), reflecting substantial differences in the transport rates of localized heat generated by the non‐radiative transitions of activated Ce^3+^ centers. The probability of non‐radiative transitions increases as the temperature rises, ultimately leading to variations in the thermal quenching resistance of the two color converter form.^[^ ^38^, ^39^ ^]^ Accordingly, from the comparison of the temperature‐dependent relative luminescent intensity between the LCMS: Ce ceramic and LCMS: Ce @ Sapphire composites, it can be demonstrated that the proposed composite exhibits superior thermal quenching robustness compared to the traditional ceramic (Figure 1h). At 150 °C, the luminescence loss of the LCMS: Ce @ Sapphire is only 15%, significantly lower than the 22% loss observed for the ceramic form. Remarkably, the composite retains 75% of its luminescent intensity even at 200 °C. Moreover, temperature‐dependent IQE measurements further confirm that the LCMS: Ce @ Sapphire composites provide enhanced thermal quenching resistance (Figure 1h).

**Figure 2:**
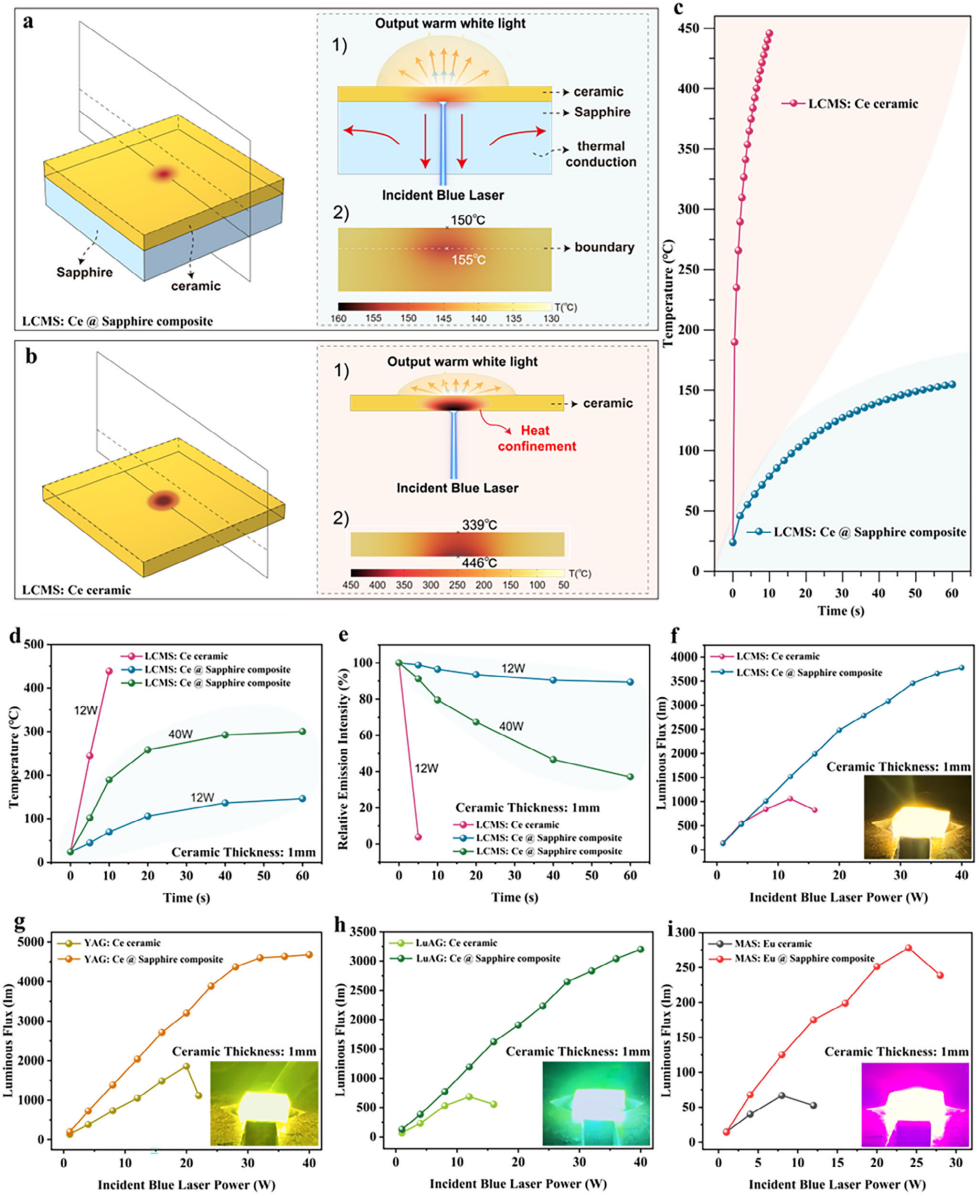
Theoretical simulations and universal applicability. Simulated temperature distribution fields of a) LCMS: Ce @ Sapphire composite and b) LCMS: Ce ceramic under blue laser power irradiation (4.72 W mm^−2^) for 60 s. c) Simulated maximum temperatures of LCMS: Ce ceramic and LCMS: Ce @ Sapphire composite under varying laser excitation times. d) Real‐time monitored local temperature at laser spot and e) corresponding relative emission intensities of LCMS: Ce @ Sapphire composite and LCMS: Ce ceramic under blue laser irradiation. The LF of f) yellow‐orange‐emitting Lu_2_CaMg_2_Si_3_O_12_: Ce^3+^ @ Sapphire, g) yellow‐emitting Y_3_Al_5_O_12_: Ce^3+^ @ Sapphire, h) green‐emitting Lu_3_Al_5_O_12_: Ce^3+^ @ Sapphire, and i) red‐emitting Mg_2_Al_4_Si_5_O_18_: Eu^2+^ @ Sapphire under various blue laser excitation; insets are the corresponding appearance under blue laser light irradiation.

Further, in order to evaluate the influence of the proposed composite material architecture on the heat dissipation performance of laser‐driven photonic converters, theoretical simulations were performed using the COMSOL Multiphysics software,^[^ ^40^ ^]^ employing a blue laser power density of 4.72 W mm^−2^ (**Figure** **2**a,b). The boundary conditions for the thermal simulations, derived from LCMS: Ce ceramic, sapphire, and LCMS: Ce @ sapphire composites, are depicted in Figure S7 (Supporting Information). Two types of photonic converters were designed for comparative analysis: the ceramic‐only converter and the ceramic@sapphire converter, with the laser beam directed at their central regions. The peak temperatures of both converters were observed at the center of the laser spot, aligning with the spatial distribution of the blue LD. Considering the laser excitation time, the localized maximum temperatures of both photonic converters increased, but remained relatively stable after 20 s of laser irradiation for ceramic@sapphire (Figure 2c). Notably, the operating temperature of the ceramic@sapphire converter remained below 155 °C, demonstrating an enhanced reduction rate of temperature due to the superior heat dissipation properties enabled by the proposed material design. In contrast, the ceramic‐only converter exhibited an operating temperature of ≈446 °C. From the perspective of theoretical simulation, these results reveal that the ceramic@sapphire configuration effectively mitigates thermal accumulation in laser‐driven photonic converters, thus making them endure higher laser power densities and achieve superior LF output.^[^ ^41^ ^]^

The actual luminescent behavior under blue laser excitation was investigated using a home‐built laser‐driven performance measurement platform in a transmissive mode (Figure S8 and Note S2, Supporting Information). The LCMS: 0.05 Ce @ sapphire exhibited the highest intensity, characterized by a broad emission peak at 580 nm under blue laser irradiation (Figure S9, Supporting Information), which is consistent with PL results (Figure 1f). Correspondingly, the maximal conversion efficiency (CE) reached 23.3% (Note S3 and Figure S10, Supporting Information and calculated using Equation 1).

(1) $$CE=\frac{P_{em}}{P_{ab}}=\frac{P_{em}}{P_{in}-P_{re}}$$

where *P_in_* and *P_re_* represent the incident blue laser optical power recorded by laser power meter and the remained blue light optical power collected by integrating sphere, respectively. Because the optical response of integrating sphere had been calibrated by using a standard Tungsten Halogen lamp, the integrations of blue bands and emission bands represent the real optical powers, corresponding to *P_re_* and *P_em_*, respectively. The luminance saturation threshold for the LCMS: Ce ceramic was observed at 12.0 W (4.72 W mm^−2^), with an LF of 1059 lm; instead, due to the innovative composite material architecture, the LCMS: Ce @ sapphire demonstrates admirable luminescent properties, exhibiting no significant luminance saturation even at the maximum laser power of 40.0 W (15.75 W mm^−2^, the upper limit of used blue laser), and achieving an LF of 3778 lm (Figure 2f). These results represent a remarkable 257% enhancement compared to the LCMS: Ce ceramic. Additionally, thermal imaging reveals significantly lower surface temperatures for the LCMS: Ce @ sapphire composite. As shown in Figure 2d,e, the LCMS: Ce ceramic experiences a substantial temperature rise after 10 s laser irradiation, reaching 439 °C @ 12.0 W (4.72 W mm^−2^), where its integrated intensity diminishes to ≈2.5%. In contrast, the proposed material architecture enabled effective thermal management, with the LCMS: Ce @ sapphire maintaining surface temperatures of only 146 °C at 12.0 W (4.72 W mm^−2^) and 300 °C at 40.0 W (15.75 W mm^−2^) during continuous blue laser irradiation for 60 s (Figure S11, Supporting Information). Facilitated by the proposed material architecture, the thermal conductivity and luminescent performance of the yellow‐orange‐emitting LCMS: Ce @ sapphire are notably superior to most of other reported high‐performance yellow‐orange‐emitting photonic converters (**Table** **1**).

**Table 1:** The achieved thermal conductivity and luminescent performance of high‐performance yellow‐orange‐emitting photonic converters upon high‐power blue laser irradiation.

Laser‐Driven photonic converter	Thermal conductivity [W m K]	Test mode	Luminance saturation [W mm^−2^]	Luminous efficiency [lm W^−1^]	Peak [nm]	Refs.
Al_2_O_3_‐(Gd,Ce)_3_(Al,Ga)_5_O_12_: Ce	13.2	reflection	6.94	115.1	576	[44]
Lu_3_MgAl_3_SiO_12_: Ce	3.57	reflection	14.5	390	564	[45]
(Lu,Gd)_3_(Sc,Al)_5_O_12_: Ce	‐	reflection	>15.7	74.6	585	[46]
YAG: Ce	‐	reflection	4	∼200	576	[47]
Lu_2_Mg_2_Al_2_Si_2_O_12_: Ce	‐	reflection	‐	81	575	[35]
(Lu,Gd)_3_Al_5_O_12_‐Al_2_O_3_: Ce	‐	transmission	>5	123	570	[48]
LCMS: Ce @ sapphire	36.9	transmission	>15.7	148	580	This work

To be noted, this novel composite architecture exhibits universal applicability across a wide range of phosphor systems. A series of composites incorporating yellow‐emitting Y_3_Al_5_O_12_: Ce^3+^, green‐emitting Lu_3_Al_5_O_12_: Ce^3+^, and red‐emitting Mg_2_Al_4_Si_5_O_18_: Eu^2+^ were synthesized (Figure S12, Supporting Information), maintaining transparency (Figures S13–S17, Supporting Information).^[^ ^42^, ^43^ ^]^ As expected, the laser‐driven luminescence performance of all the composites was markedly enhanced by the proposed material architecture. The LF demonstrated exceptional 152%, 319%, and 316% improvements for the yellow‐, green‐, and red‐emitting composites, respectively, while the luminance saturation threshold increased to a factor of 2–3, collectively revealing the effectiveness and versatility of this architecture in enhancing luminescence performance (Figure 2g–i).

**Figure 3:**
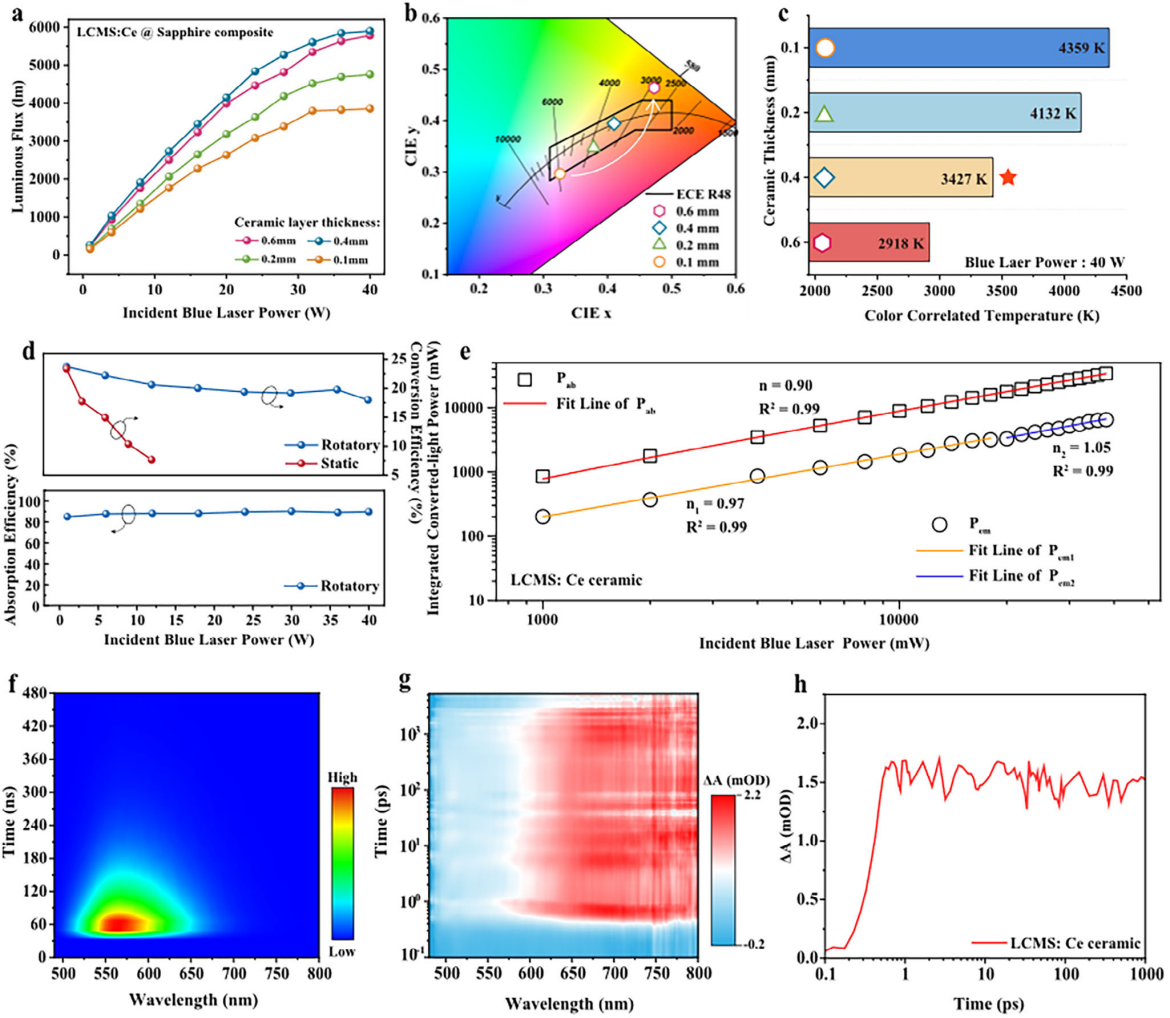
Regulation of Photometric/chromatic parameters and luminance saturation mechanism. a) LF, b) chromaticity coordinates, and c) CCT of LCMS: Ce @ sapphire composites with different phosphor ceramic layer thicknesses. d) Absorption efficiencies under static excitation (lower graph) and conversion efficiencies under static/rotatory excitation (upper graph) for LCMS: Ce ceramic. e) Fitting lines of the absorbed blue lasers power (P_ab_), and the emitted light power (P_em_) of LCMS: Ce ceramic. f) Time‐resolved photoluminescence (TRPL) spectra of LCMS: Ce ceramic. g) Contour plot of the *fs* TA spectra of LCMS: Ce ceramic upon photoexcitation at 450 nm with a time window from 0 to 8000 ps. h) Corresponding ultrafast dynamics curves probed at 650 nm.

Taking LCMS: Ce @ sapphire composite as an example, precise control over the thickness of the phosphor ceramic layer is required to achieve high‐quality laser‐driven lighting with desirable photometric/chromatic parameters. Under 40 W (15.75 W mm^−2^) blue laser excitation, no significant luminance saturation is observed regardless of the thickness of the ceramic layer. Evidently, the LCMS: Ce @ sapphire composite demonstrates excellent luminescent properties, delivering LF in the range of 3852–5902 lm and LE between 96 and 148 lm W^−1^ (**Figure** **3**a; Figure S18, Supporting Information). By varying the thickness of the phosphor ceramic layer, three variants of LCMS: Ce @ sapphire (with ceramic layer thicknesses of 0.1, 0.2, and 0.4 mm) are shown to meet the ECE R48 standard (European requirements for the lighting devices on vehicles) (Figure 3b). Correspondingly, as the ceramic thickness increases, the correlated color temperature (CCT) becomes progressively warmer, decreasing from 4359 K to 2918 K (Figure 3c). Upon comprehensive consideration of LF, LE, chromaticity coordinates and CCT, the optimized composite is identified as LCMS: Ce @ sapphire with a phosphor ceramic layer thickness of 0.4 mm. This optimized composite achieves an LF of 5902 lm @ 40 W (15.75 W mm^−2^), an LE of 148 lm W^−1^, chromaticity coordinates of (0.4101, 0.3944), color rendering index (CRI) of 60, and a CCT of 3427 K, aligning with the near warm white light specification outlined in the Chinese GB50034‐2013 standard. Further, the LCMS: Ce @ sapphire composite retains uniform angular color distribution and exhibits superior light uniformity, as schematically illustrated in Figures S19–S21 (Supporting Information).

Typically, for laser‐driven photonic converters, notorious luminance saturation arises from the combined effects of thermal quenching (thermal saturation) and optical excitation intensity quenching (optical saturation). In static excitation, thermal saturation and optical saturation coexist, while optical quenching dominates in rotational excitation. As shown in Figure 3d **(upper)**, the conversion efficiency in rotational mode consistently exceeds that in static mode, thus making thermal and optical saturation contribute 83.5% and 16.5% to luminance saturation, respectively (Note S4, Supporting Information). Regarding optical saturation, from the perspective of photon numbers, the saturation of optical intensity can be composited of the single‐photon and the double‐photon processes under high‐power blue laser irradiation (Figure 3e; Note S5, Supporting Information and Equation 2).^[^ ^20^, ^49^ ^]^

(2) lg(I)=nlg(P)

where n denotes the number of photons required to emit a single photon, I represents the luminous intensity, and P refers to the driving power, with n determined using the slope method. Time‐resolved photoluminescence (PL) spectra confirm a single type of Ce^3+^ luminescence center (Figure 3f). As shown in Figure 3d **(lower)**, a nearly constant absorption efficiency (< 5%) demonstrates sufficient electrons in the ground state for excitation and ruling out the possibility of ground state depletion (GSD). The femtosecond transient absorption (fs‐TA) spectra of LCMS ceramics upon photoexcitation at 450 nm show an excited‐state absorption signal in the spectral range of ∼590–800 nm (Figure 3g). The overlap between the absorption band and the emission spectra indicates photon reabsorption effect:^[^ ^1^ ^]^ emitting photons can be reabsorbed by excited Ce^3+^, leading to the electron excitation from the excited state to the conduction band through an excited state absorption “upconversion” (ESA) process, and^[^ ^2^ ^]^ Förster‐Dexter resonance energy transfer induces a strong energy‐transfer “upconversion” effect, where interactions between two adjacent excited‐state Ce^3+^ ions lead to the emission of a single photon at the expense of multiple photons (Auger energy‐transfer “upconversion”, ETU).^[^ ^13^, ^50^ ^]^ The two processes both contribute to a nonlinear relationship between emission intensity and excitation power, further resulting in optical saturation. Additionally, the ultrafast dynamics curve (Figure 3h) probed at 650 nm demonstrates that excited‐state absorption (ΔA > 0) occurs instantaneously, increasing rapidly to its maximum value within a very short time (∼0.35 ps) and accompanied by a fast luminescence process lasting up to the detection limit (∼8000 ps). The sufficient electrons are “stored” in the excited state, enabling their subsequent transport to the conduction band. Obviously, since optical saturation is an intrinsic and unavoidable property, effective thermal management is essential for optimizing laser‐driven photonic converters, particularly through innovative material architectures.

**Figure 4:**
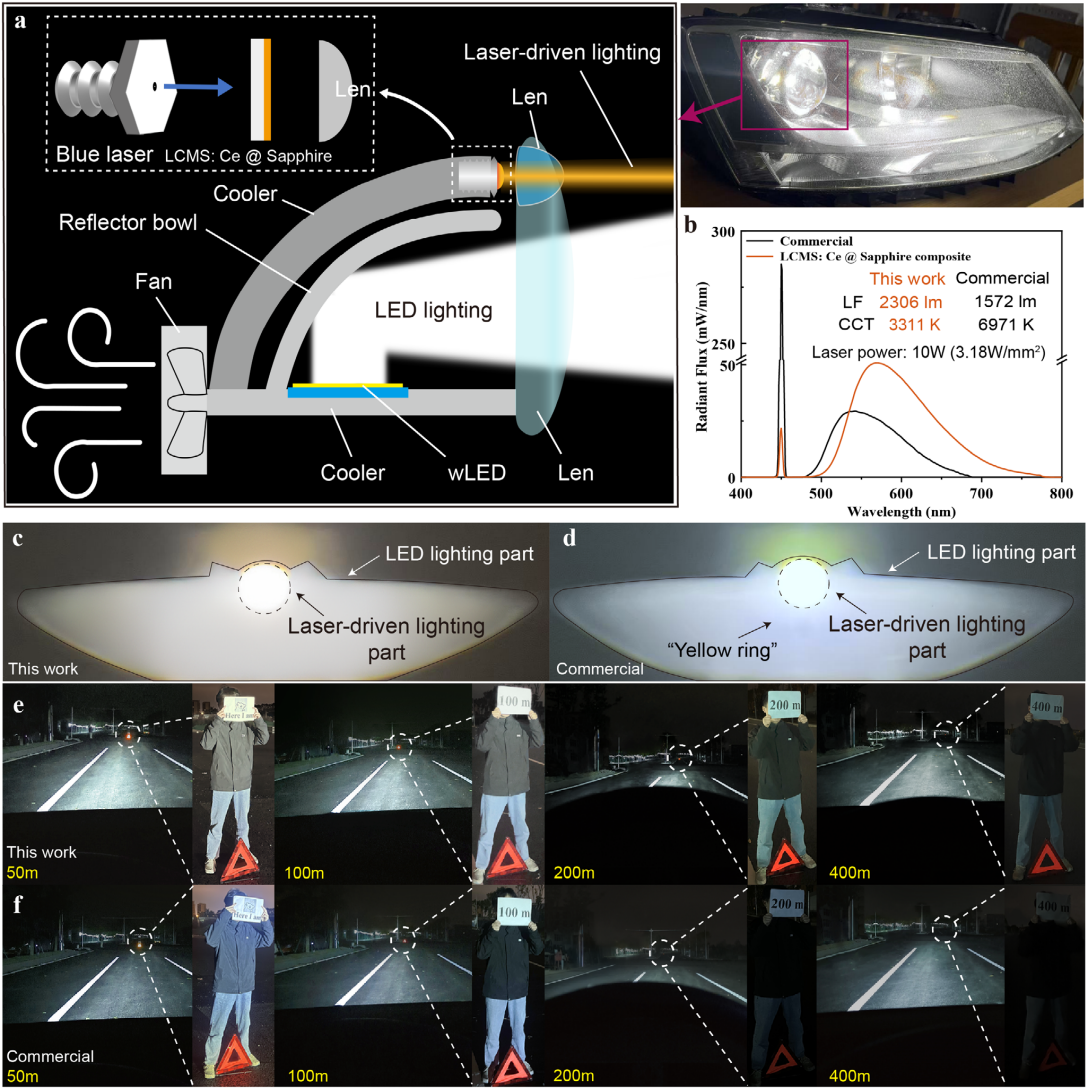
Demonstration experiments for car headlight device. a) Schematical illustration of the car headlight device based on a 450 nm blue LD and LCMS: Ce @ sapphire composite. b) Comparison of EL spectra and c,d) color distribution for the LCMS: Ce @ sapphire‐based car headlight prototype and a commercial headlight device. e,f) Actual illumination effects of LCMS: Ce @ sapphire‐based car headlight prototype and commercial headlight device at varying distances during nighttime driving.

With the rapid development of new energy vehicles, laser‐driven headlights have emerged as a highly promising and advanced lighting technology with significant demands. In order to evaluate the feasibility of practical applications, a car headlight device based on a 450 nm blue LD and newly proposed color converters was constructed (**Figure** **4**a). Notably, compared to commercial counterpart, the electroluminescence (EL) spectra of the LCMS: Ce @ sapphire‐based system exhibit significantly higher luminescent intensity, attributing to the employed novel structural strategy (Figure 4b). Moreover, the LCMS @ Sapphire ceramic‐based design demonstrates a homogeneous color distribution (center: 3685 K, edge: 3620 K), in stark contrast to the commercial alternative (center: 8544 K, edge: 4640 K) that suffers from the common issue of a “yellow ring” (Figure 4c,d; Figure S22, Supporting Information). Additionally, the LCMS: Ce @ sapphire‐based demo achieves superior light collimation and an extended illumination distance of over 400 m, outperforming conventional models (Figure 4e,f; Figure S23, Supporting Information). The nearly warm white light produced, enriched with red light, is less prone to scattering, making it particularly well‐suited for long‐distance illumination under bad weather conditions such as rain or fog. Evidently, the proposed material architecture represents an effective strategy for enhancing the performance of color converters, paving the way for next‐generation long‐distance lighting systems.

## 3 Discussion

This study proposes a facile and scalable photonic converters architecture, ceramic@sapphire composites. Theoretical and experimental studies reveal that the strong interfacial bonding between the phosphor ceramic layer and sapphire substrate ensures efficient heat dissipation and enhanced luminescence properties. The universality of this strategy is confirmed across various phosphor systems, with significant enhancements in luminescence efficiency and saturation thresholds. The resultant LCMS: Ce @ sapphire composite exhibits luminous flux of 5902 lm, luminous efficacy of 148 lm W^−1^, warm CCT of 3427 K, and desired CRI of 60, together with superior thermal quenching resistance, retaining 75% of its luminescent intensity at 200 °C. Practical implementation in automotive headlights highlights its superior performance, including enhanced luminance intensity, extended illumination range, and uniform color distribution. This study offers a straightforward and generalizable approach to high‐performance photonic converters, expanding the possibilities for next‐generation laser‐driven lighting applications.

## 4 Experimental Section

### Preparation of LCMS Ceramic Layer

The Lu_2‐x_CaMg_2_Si_3_O_12_: xCe^3+^ (x = 0.01–0.09) ceramic layer were fabricated by a high‐temperature solid‐state reaction. Commercial oxide powders of Lu_2_O_3_ (99.99%), CaCO_3_ (99.99%), MgO (99.99%), SiO_2_ (99.99%), and CeO_2_ (99.99%) were the initial raw materials and weighed according to the chemical stoichiometry. Then, the mixed powders were transferred into crucible after grounding together for 15 min. The samples were placed in a furnace under a flowing H_2_/N_2_ (10%/90%) gas, heated at a rate of 10 °C min^−1^ to 1590 ° C, held for 2 h, then cooled down to 500 °C at the same rate of 10 °C min^−1^, followed cooling with furnace to room temperature. The obtained ceramics were cut and polished to get a flat surface.

### Preparation of Ceramic@Sapphire

A sapphire substrate was placed on top of the synthesized ceramic layer surface and thermally coupled with the ceramic under specific conditions. The coupling process was conducted at 1400 °C for 2 h to obtain yellow‐orange‐emitting LCMS: Ce @ sapphire, at 1775 °C for 2 h for yellow‐emitting Y_3_Al_5_O_12_: Ce^3+^ @ sapphire, at 1830 °C for 2 h for green‐emitting Lu_3_Al_5_O_12_: Ce^3+^ @ sapphire, and at 1550 °C for 10 min for red‐emitting Mg_2_Al_4_Si_5_O_18_: Eu^2+^ @ sapphire in vacuum furnace. The temperature was increased at a rate of 10 °C min^−1^ up to 1300 °C, followed by a slower rate of 3 °C min^−1^ beyond 1300 °C. After integration process, the furnace was cooled at 5 °C min^−1^ down to 300 °C, and then cooled to room temperature. Finally, the ceramic@sapphire composites were cut into square pieces for subsequent characterization.

### Characterization

The microstructure and elemental distribution of LCMS: Ce @ sapphire were analyzed using a COXEM EM‐30 scanning electron microscope (SEM) equipped with an energy‐dispersive spectrometer (EDS). Phase identification was performed using an X‐ray powder diffractometer (SmartLab SE, Rigaku) with a Cu Kα radiation source, operating at a voltage of 40 kV. The diffraction patterns were recorded in the range of 2θ = 10°–80° with a scanning rate of 10° min^−1^. Photoluminescence (PL) and photoluminescence excitation (PLE) spectra, decay curves, and photoluminescence quantum yield (PLQY) measurements were conducted using a fluorescence spectrometer (FLS1000, Edinburgh Instruments, UK). For PLQY determination, samples were positioned inside a barium sulfate‐coated integrating sphere with a diameter of 15 cm, integrated with the spectrometer. Photometric and colorimetric parameters of the samples under blue laser excitation were obtained using a home‐made laser illumination system consisting of a 30 cm integrating sphere (Labsphere, Inc., USA), a blue laser source (LSR‐PS‐FA, Lasever, China), and a calibrated CCD spectrometer (OHSP‐350 M, Hopoocolor Technology Co., Ltd., China). The laser spot diameter was adjusted to 2.54 mm^2^ using a focusing lens (Figure S24, Supporting Information). The surface temperature of the samples was measured using an infrared camera (TiS75, Fluke, USA), positioned 10.0 cm from the sample with proper focus adjustment. Thermal conductivity was assessed using a laser flash thermal conductivity analyzer (NETZSCH LFA 457).

## Conflict of Interest

The authors declare no conflict of interest.

## Author Contributions

G.Y.X. and S.S.L. contributed equally to this work. S.S.L. conceived the composite design. G.Y.X. synthesized the material and wrote the first draft. S.S.L. and D.Q.C. helped to analyze the experimental results and finish the final manuscript. T.J.S., Y.X.K., Z.H.Z., and R.H.C. helped to measure the spectroscopy. Z.K.Y. helped to make computational procedures. Y.P., T.P., L.W.Z., and F.H. provided constructive suggestions to data analyses. D.Q.C. supervised the project.

## Supporting information



Supporting Information

## Data Availability

The data that support the findings of this study are available from the corresponding author upon reasonable request.
