# Supplementary material for: Transparent Ceramic@Sapphire Composites for High‐Power Laser‐Driven Lighting
Source: Adv Sci (Weinh). 2025 Apr 29;12(27):2505232. doi: 10.1002/advs.202505232 (PMC12279172; doi:10.1002/advs.202505232)
Supplement: Supplementary file 1 — Supporting Information [file ADVS-12-2505232-s001.docx]

**Supporting Information**

**Transparent Ceramic@Sapphire Composites for High-Power Laser-Driven Lighting**

Guoyu Xi ^1,^ *^+^*, Shisheng Lin ^1,^ *^+^* ^*^, Tongjie Shen ^1^, Tao Pang ^2^, Zikang Yu ^3^, Yang Peng ^3^, Lingwei Zeng ^4^, Yuxiang Ke ^1^, Zhehong Zhou ^1^, Ronghua Chen ^1^, Feng Huang ^1^, Daqin Chen ^1, 5, 6, *^

[1] G. Y. Xi, Dr. S. S. Lin, T. J. Shen, Y. X. Ke, Z. H. Zhou, R. H. Chen, Prof. F. Huang, Prof. D. Q. Chen

College of Physics and Energy, Fujian Normal University, Fuzhou, Fujian, 350117, P. R. China

E-mail: linshisheng@fjnu.edu.cn (S. S. Lin); dqchen@fjnu.edu.cn (D. Q. Chen)

[2] Dr. T. Pang

Huzhou Key Laboratory of Materials for Energy Conversion and Storage, College of Science, Huzhou University, Huzhou, Zhejiang, 313000, P. R. China

[3] Z. K. Yu, Prof. Y. Peng

School of Mechanical Science and Engineering, Huazhong University of Science and Technology, Wuhan, Hubei, 430074, P. R. China

[4] Dr. L. W. Zeng

School of Chemistry and Chemical Engineering, Hunan University of Science and Technology, Xiangtan, Hunan, 411201, P. R. China

[5] Prof. D. Q. Chen

Fujian Provincial Collaborative Innovation Center for Advanced High-Field Superconducting Materials and Engineering, Fujian Normal University, Fuzhou, Fujian, 350117, P. R. China

[6] Prof. D. Q. Chen

Fujian Provincial Engineering Technology Research Center of Solar Energy Conversion and Energy Storage, Fujian Normal University, Fuzhou, Fujian, 350117, P. R. China

^+^ G. Y. Xi and S. S. Lin contributed equally.


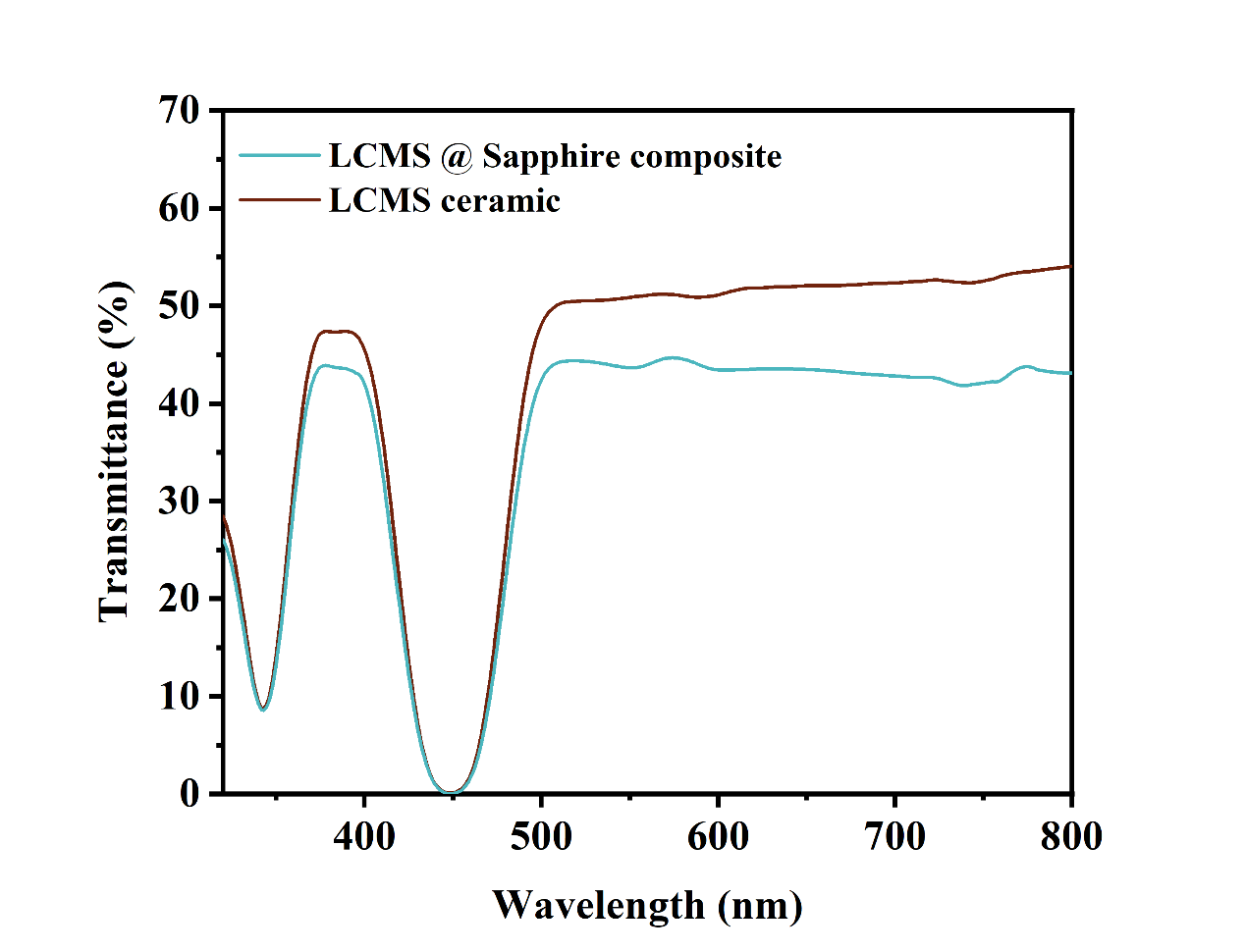


**Figure S1.** The transmittance spectra of LCMS: 0.05Ce ceramic and LCMS: Ce @ Sapphire composites.

**
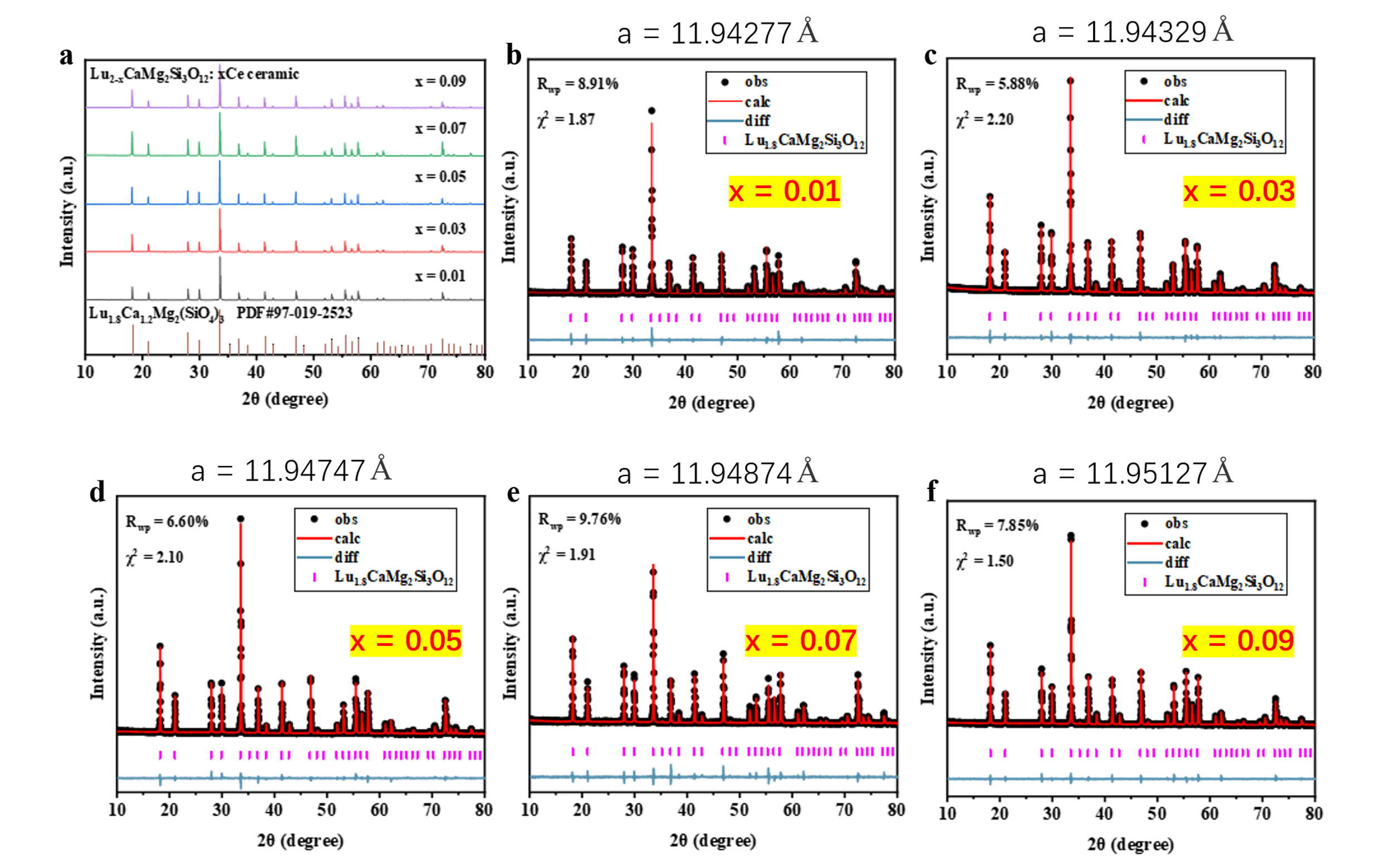
**

**Figure S2.** Rietveld refinement XRD pattern of LCMS: xCe (x = 0.01-0.09) ceramics.


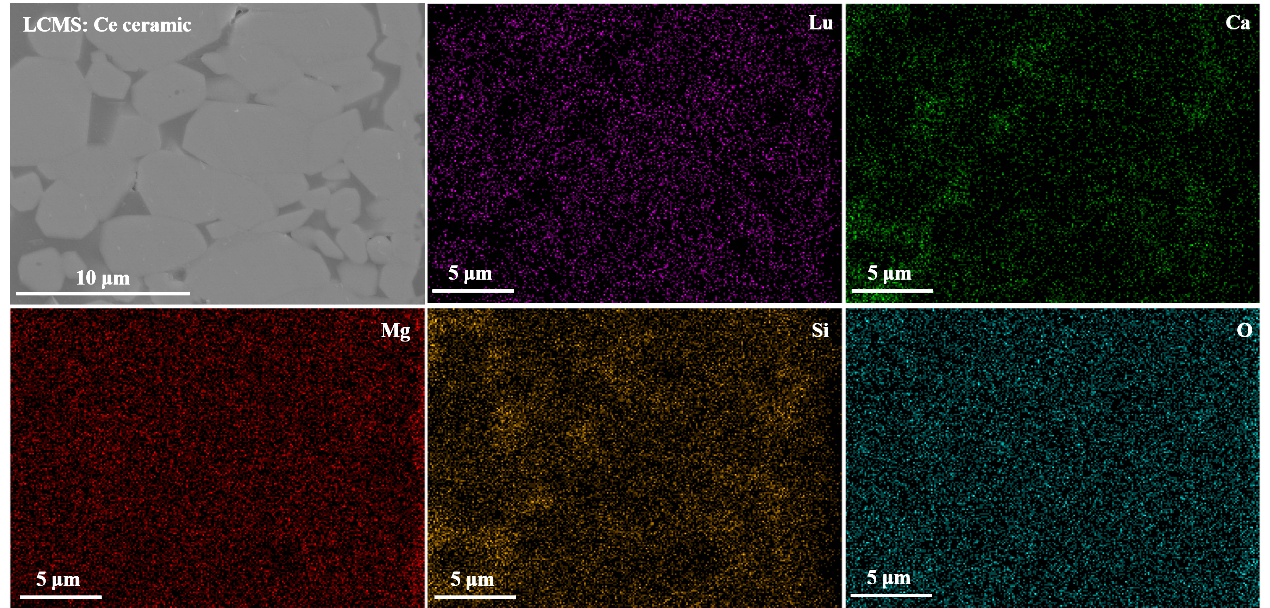


**Figure S3.** Front-view SEM and EDS mapping of the LCMS: Ce @ Sapphire composite.


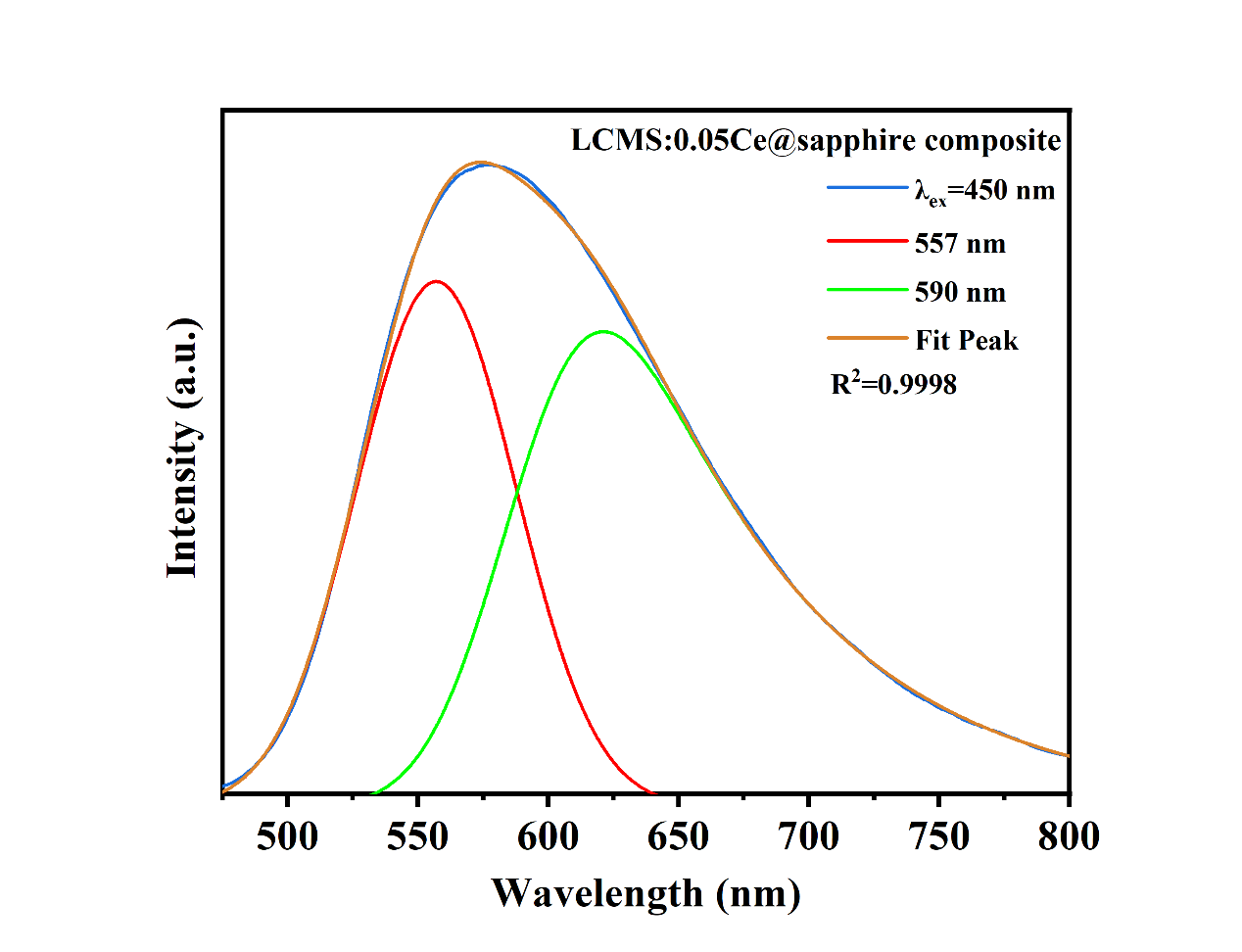


**Figure S4.** The Gaussian fitting of the emission band of LCMS:0.05Ce@sapphire composite.

**Discussions on Figure S4**

The Ce^3+^ emission observed at around 580 nm originates from the spin-allowed 5d↔4f transitions, specifically to the ^2^F_5/2_ and ^2^F_7/2_ levels. These transitions produce two overlapping emission bands, resulting in a broad emission centered near 580 nm. The emission spectrum was fitted using a double-Gaussian model. Two peaks were identified at approximately 557 nm and 590 nm with FWHM of 31 nm and 27 nm, respectively.

**
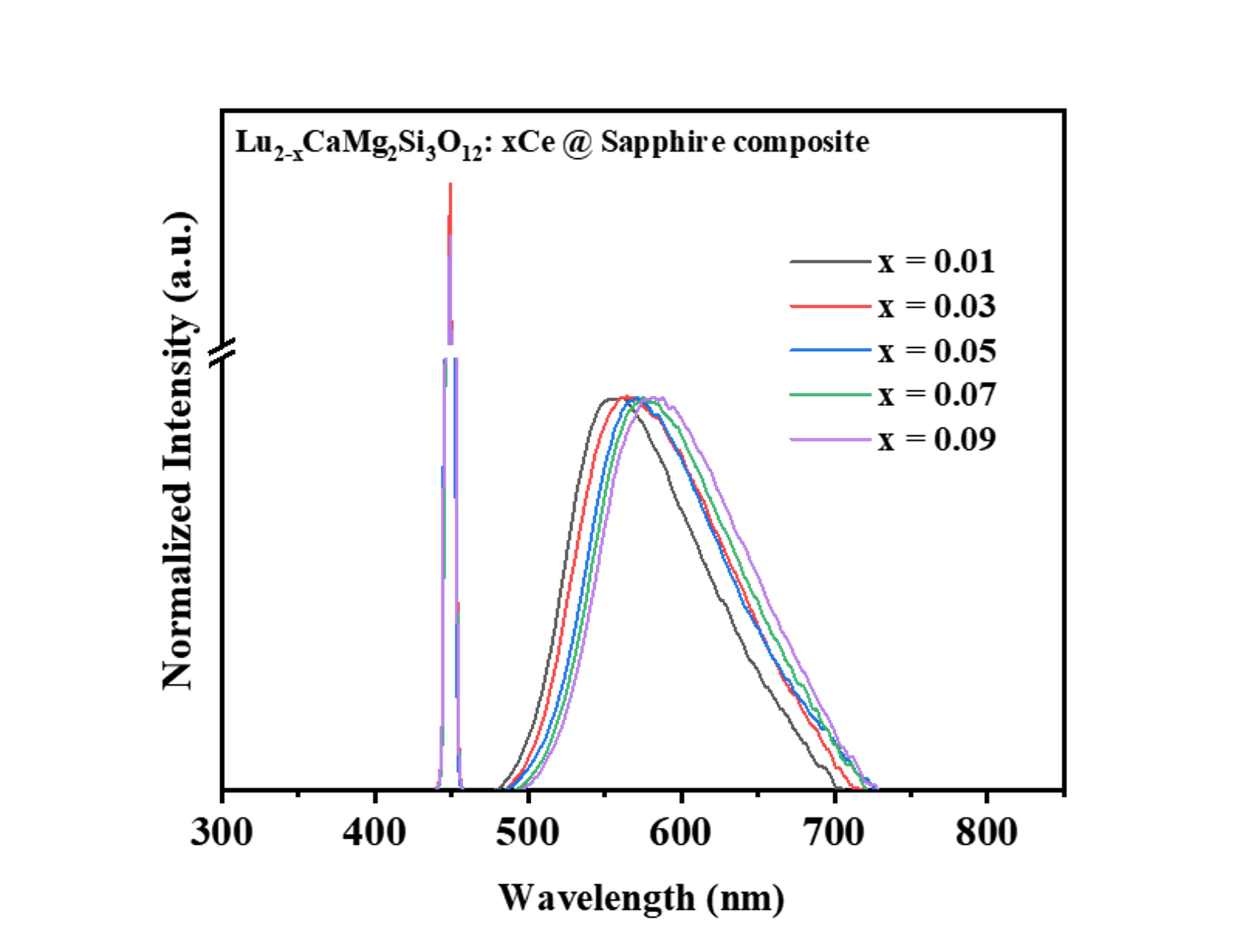
**

**Figure S5.** Normalized PL spectra of LCMS: xCe @ Sapphire (x = 0.01-0.09) under 450 nm laser excitation.

**
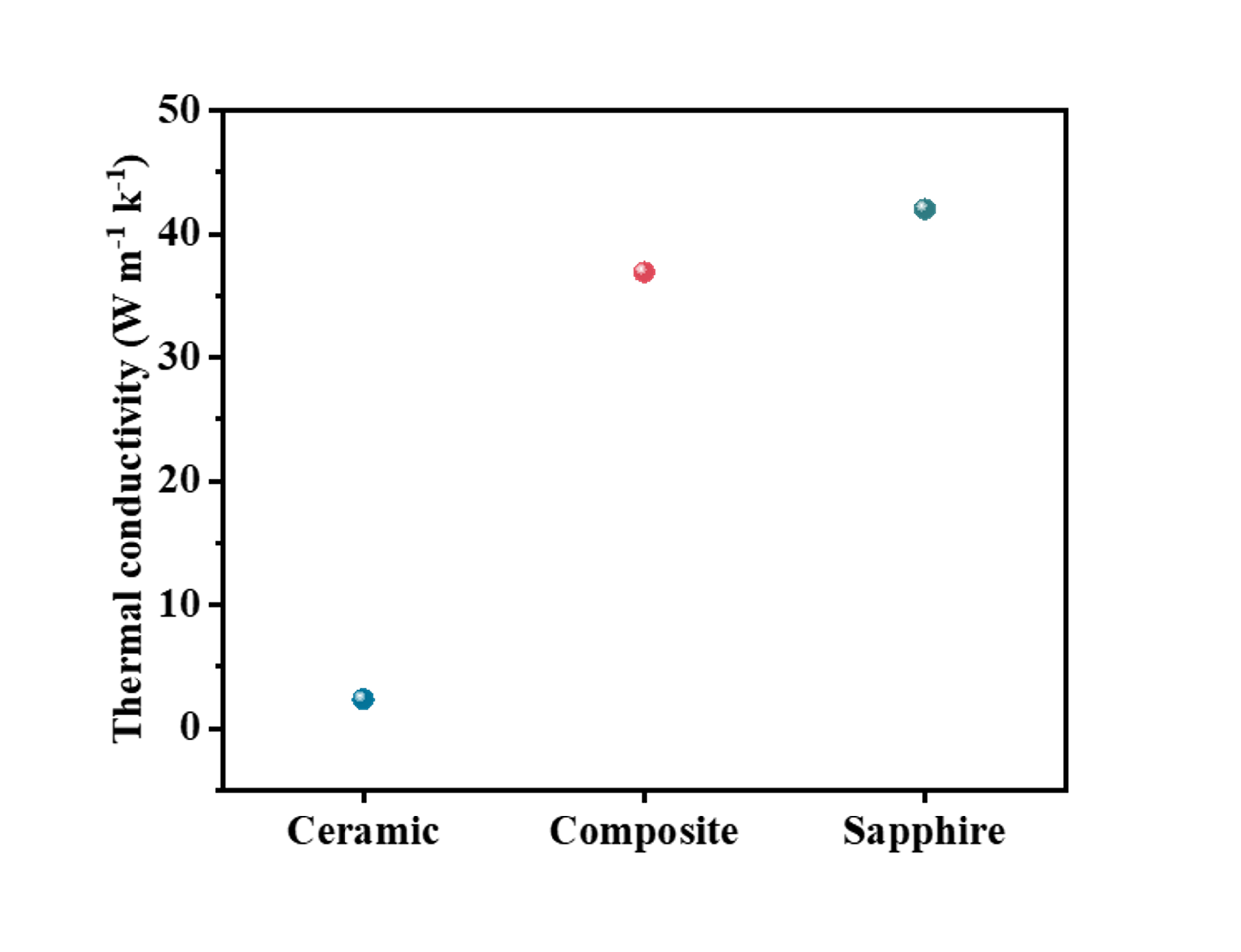
**

**Figure S6.** Thermal conductivity of LCMS: Ce ceramic, LCMS: Ce @ Sapphire composite and Sapphire (thickness: 2.0 mm).

**Discussions on Figure S6**

The heat conductivity km of multiphase composite material can be defined as follows [*Introduction to ceramics*, John wiley & sons, 1976*; J. Mater. Chem. C*, 2019, 7, 8120]*:*

$k_{m}=k_{c}\frac{1+2v_{d}(1-k_{c}/k_{d})(1+2k_{c}/k_{d})}{1-v_{d}(1-k_{c}/k_{d})(1+k_{c}/k_{d})}$ (**Equation S1**)

In which, k_c_, k_d_ and ν_d_ are the heat conductivity of continuous phase, the heat conductivity of dispersive phase and the fraction of dispersive phase, respectively. For the representative LCMS: Ce @ Sapphire composite, the coefficient of heat conductivity for LCMS: Ce ceramic and Sapphire are 2.3 W m^-1^ K^-1^ (test result), 42 W m^-1^ K^-1^ (SA100, KYOCERA) and ν_d_ is approximated to 0.167 respectively. k_m_ could be approximated to 33.9 W m^-1^ K^-1^, close to the result value. Obviously, integration with sapphire resulted the heat conductivity in a significant increase.


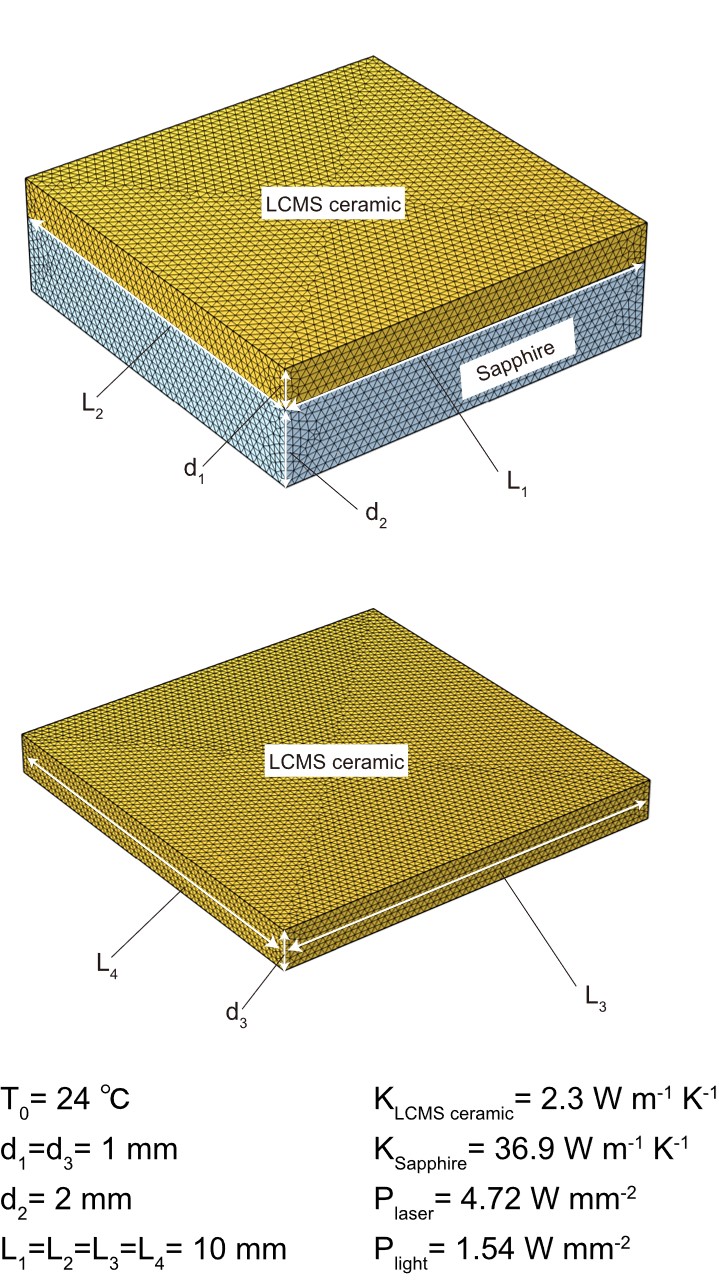


**Figure S7.** Thermal boundary conditions of LCMS: Ce @ Sapphire composite (upper), and LCMS: Ce ceramic (below).

**Discussions on Figure S7**

The heat power was calculated by the difference between laser power and light power of LCMS: Ce @ Sapphire composite and LCMS: Ce ceramic$\text{(}\text{P}_{\text{heat}}\text{ = }\text{P}_{\text{laser}} \text{-}\text{ }\text{P}_{\text{light}}\text{)}$, (*P*_laser_ = 4.72 W·mm^-2^) [*Adv. Funct. Mater.*, 2025, 35, 2414023]. The laser spot area was set as 2.54 mm^2^. Based on the product brochure and measurement of thermal conductivity, that of the Sapphire and ceramic layer was set as 42 W·m^-1^·K^-1^ and 2.3 W·m^-1^·K^-1^, respectively.

The surrounding air was held constant at 24 ℃. At the top and side surfaces, a convective heat flux boundary condition was used, which is driven by the temperature difference between the converter and the surrounding atmosphere: $q=h(\text{T}_{\text{ext}}\text{-T)}$. Here q is the inward heat power and h is the heat transfer film coefficient.


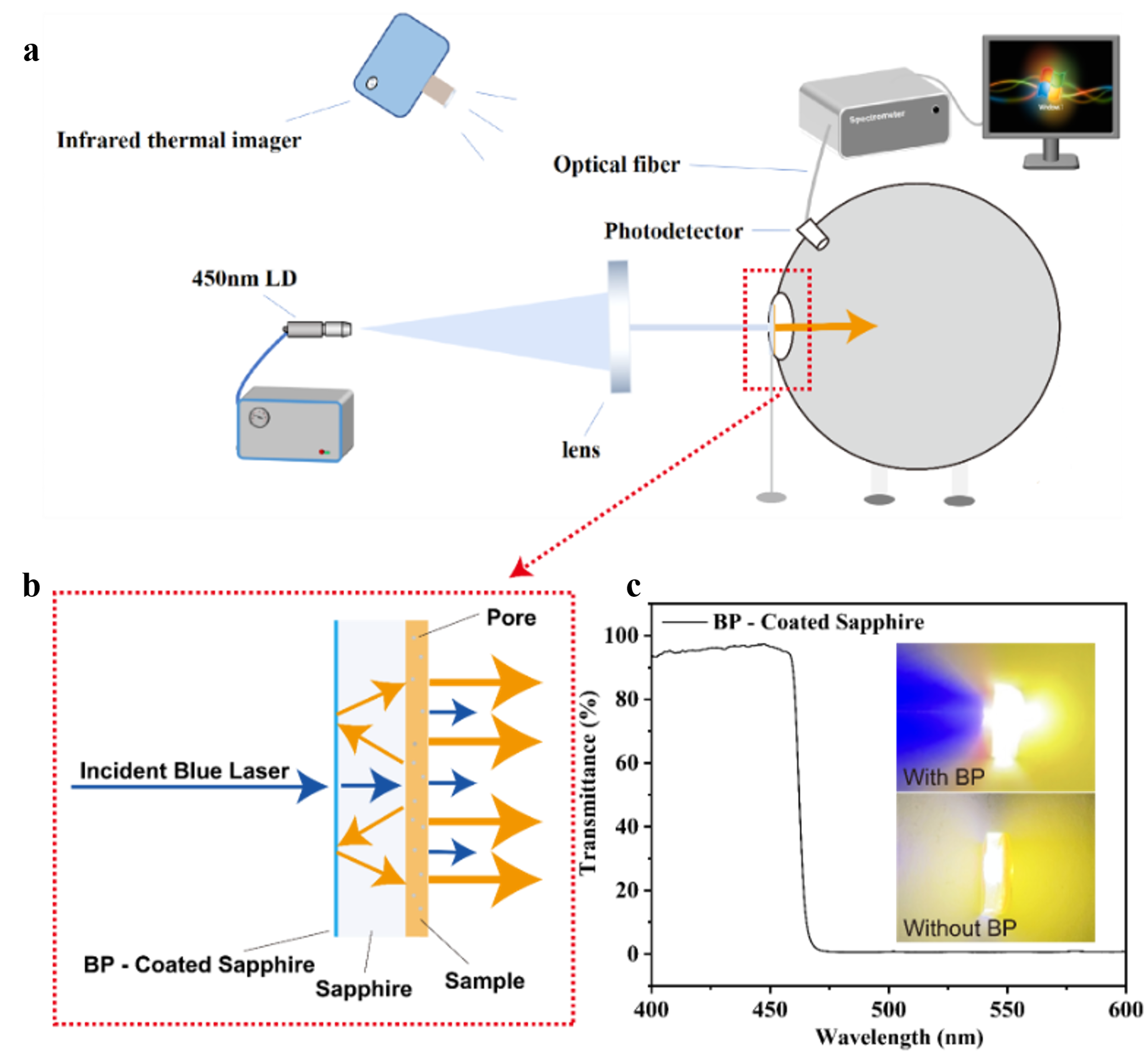


**Figure S8.** Schematical illustration of the home-built measurement platform in a transmissive mode to evaluate laser-driven performance.

**
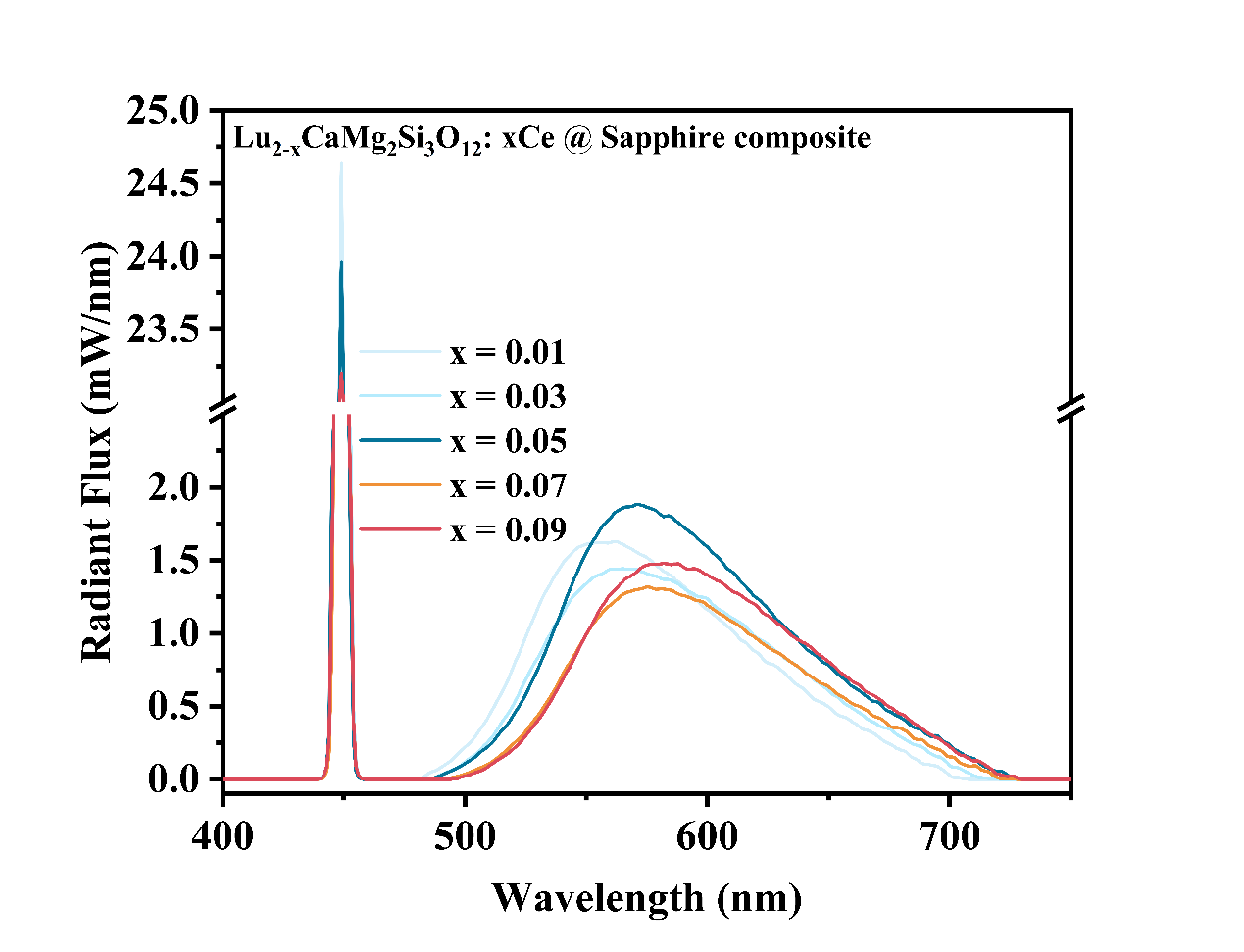
**

**Figure S9.** Electroluminescent (EL) spectra of LCMS: xCe @ Sapphire (x = 0.01-0.09) composite under 450 nm laser excitation.


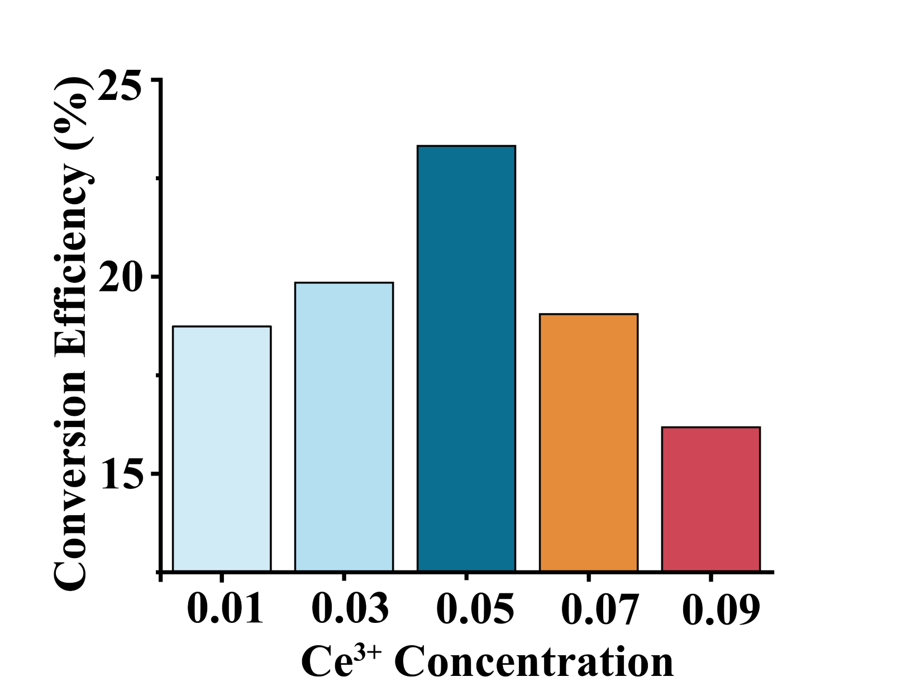


**Figure S10.** Conversion efficiency (CE) of LCMS: xCe @ Sapphire (x = 0.01-0.09) composite under 450 nm laser excitation.


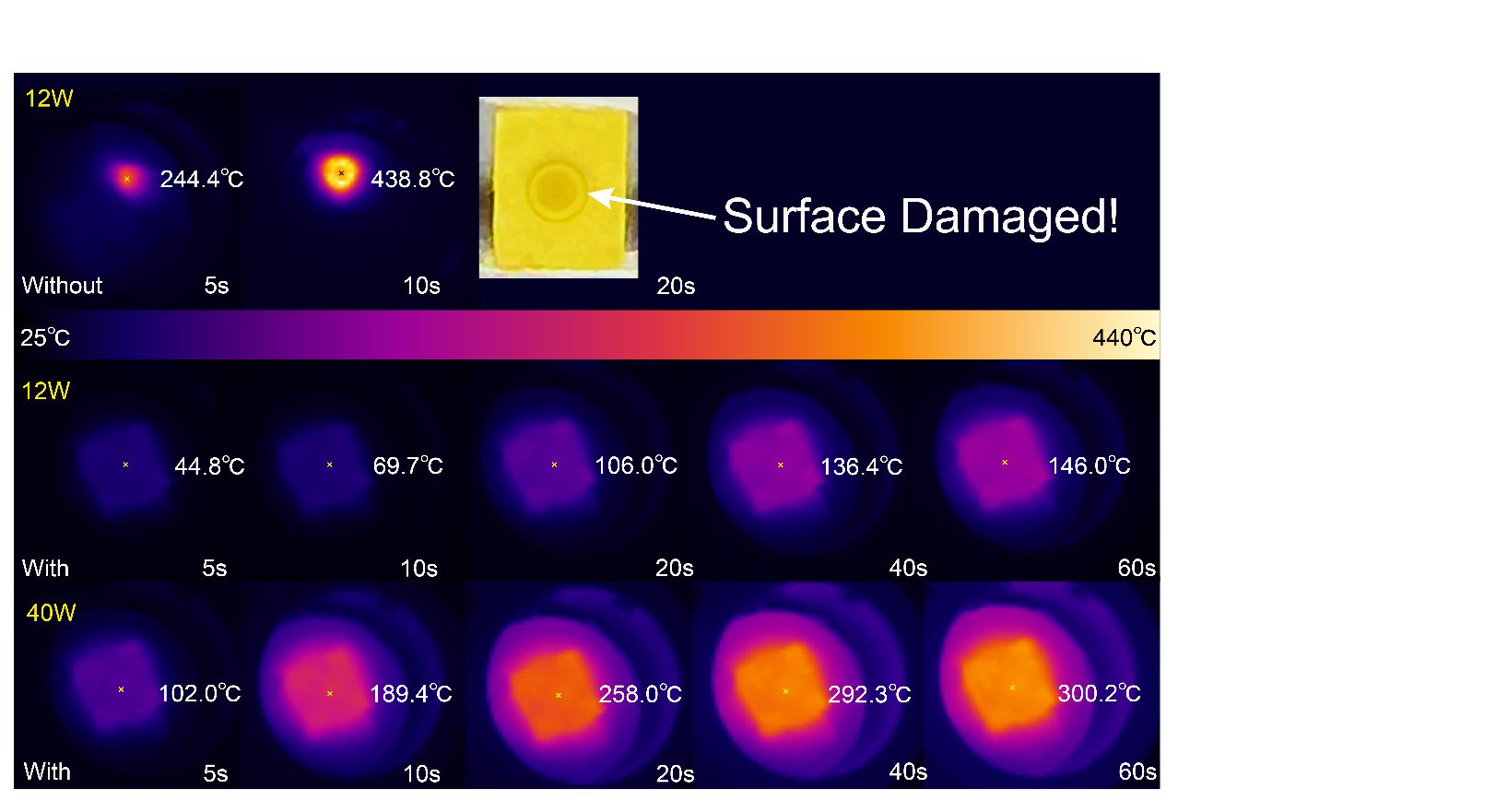


**Figure S11.** Thermal imaging photographs of LCMS: Ce ceramic and LCMS: Ce @ sapphire composites under irradiation by different power densities of a blue laser.

**Discussions on Figure S11**

Thermal imaging of the LCMS: Ce ceramic under 12W blue laser irradiation revealed a rapid temperature increase to 244.4 ℃ within 5 s (first line), followed by irreversible surface damage after only 20 s of continuous exposure. In contrast, real-time thermal imaging of the LCMS: Ce @ sapphire composites under continuous 12 W laser irradiation demonstrated a significant reduction in surface temperature from 438.8 ℃ to 69.7 ℃ within the same 10 s irradiation period, representing an 84 % decrease (second line). Furthermore, even under continuous 40W laser irradiation, the surface temperature of the composite was effectively maintained at approximately 300 ℃ after 60 s (third line). Thermal imaging photographs exhibit that the LCMS: Ce @ sapphire composites possess efficient heat dissipation ability, effectively suppressing temperature rise and thereby significantly enhancing the luminicence performance.


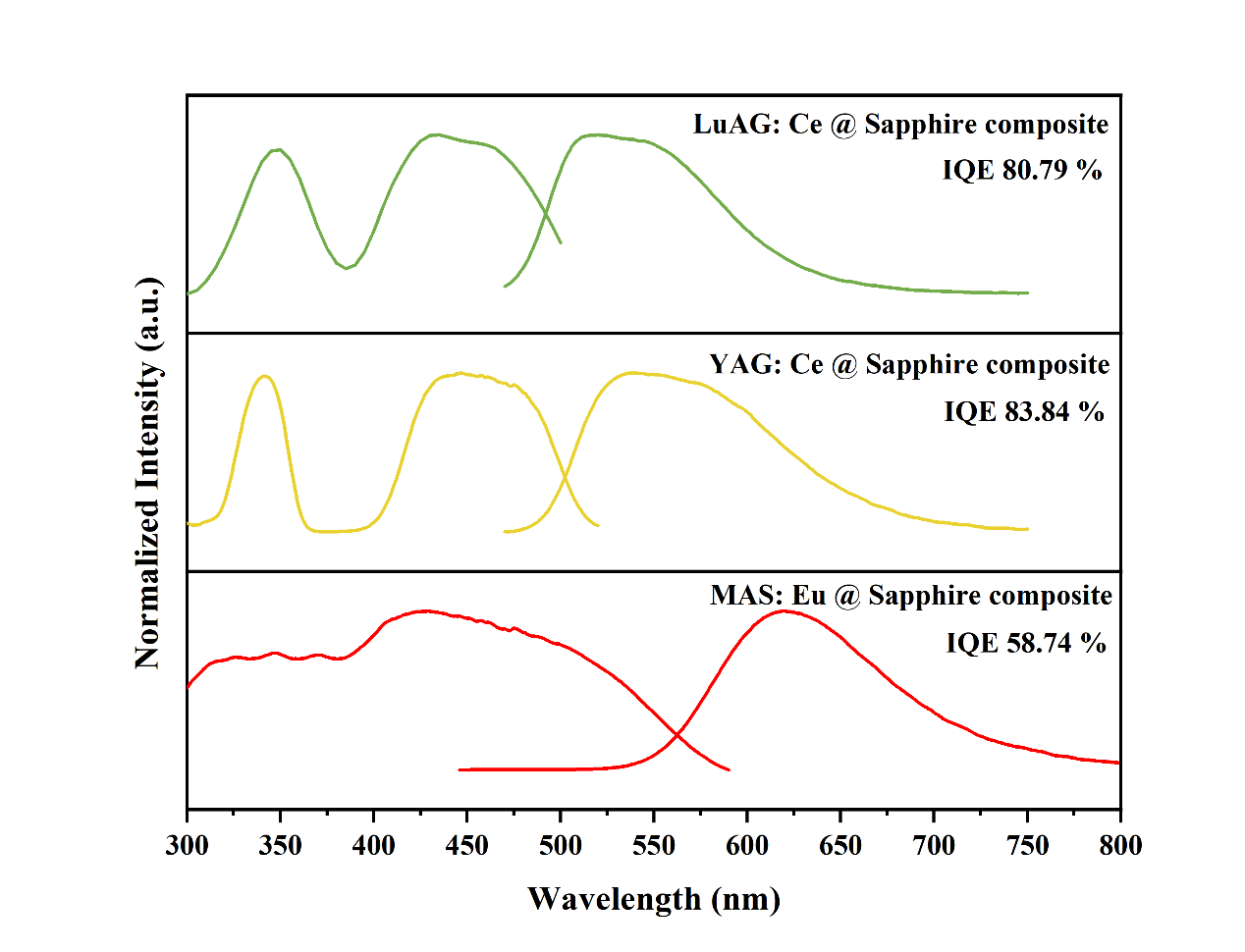


**Figure S12.** The PL/PLE spectra and the quantum efficiencies of the LuAG, YAG and MAS @ Sapphire composites

**
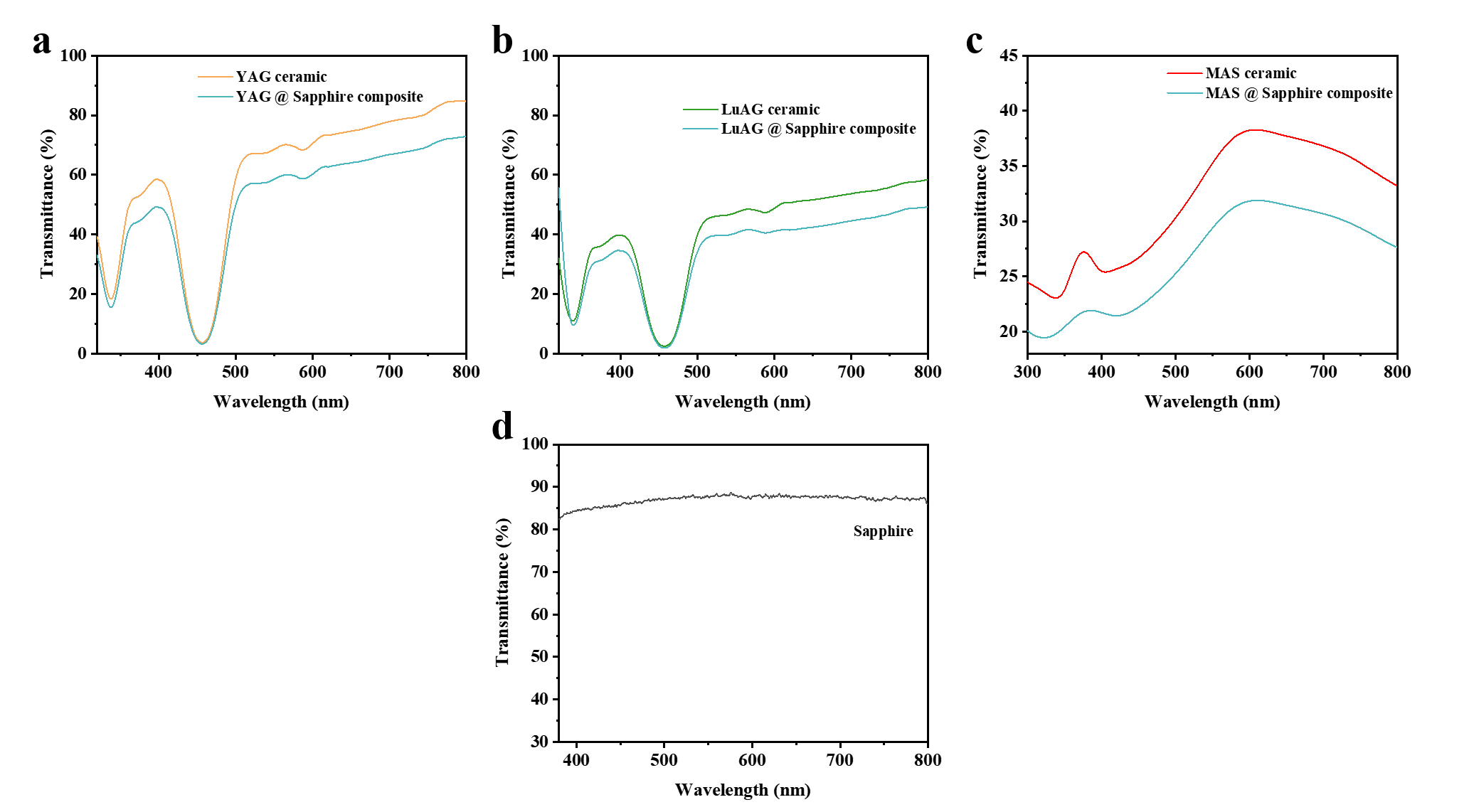
**

**Figure S13.** The comparison of the transmittance spectra between two types of photonic converters.

**Discussions on Figure S13**

All ceramic layers have a thickness of 0.4 mm, and the sapphire substrate is 2.0 mm thick. As shown in Figure S13d, the transmittance of sapphire is approximately 86%. With the integration of sapphire, the transmittance of YAG, LuAG, and MAS phosphor ceramics all decreased slightly. Specifically, for YAG @ Sapphire composite, the transmittance decreased from 67.2 % to 57.2 % @ 530 nm, corresponding to a reduction of approximately 15 %; for LuAG @ Sapphire composite, the transmittance decreased from 44.5 % to 38.3 % @ 510 nm, corresponding to a reduction of approximately 14 %; and for MAS@ Sapphire composite, the transmittance decreased from 38.3 % to 31.9 % @ 610 nm, corresponding to a reduction of approximately 17 %.

**
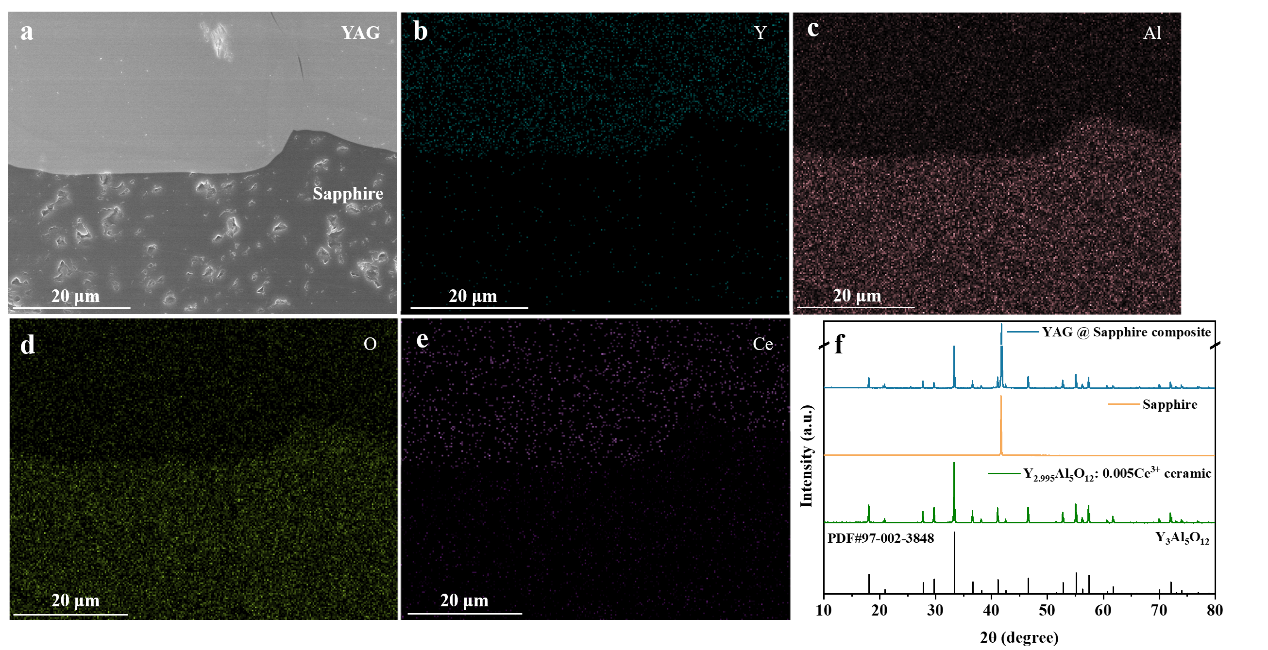
**

**Figure S14.** a) SEM, b-e) EDS mapping and f) XRD pattern of Y_3_Al_5_O_12_: Ce^3+^ (YAG: Ce) @ Sapphire composite.

**
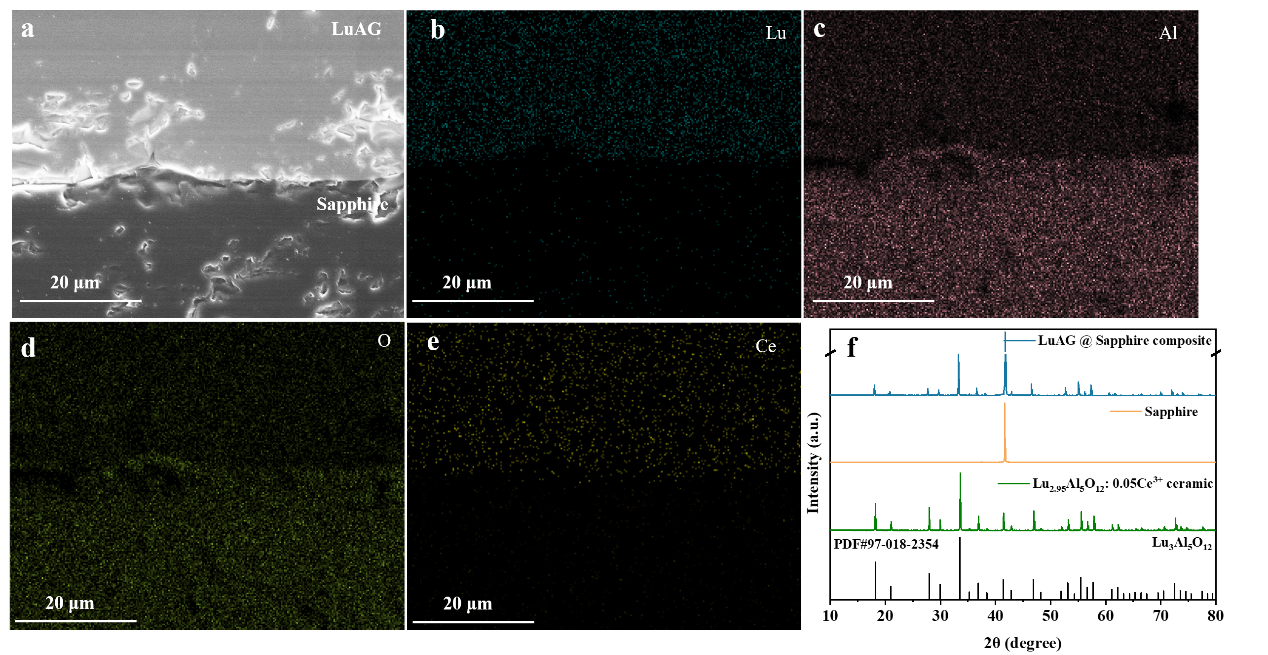
**

**Figure S15.** a) SEM, b-e) EDS mapping and f) XRD pattern of Lu_3_Al_5_O_12_: Ce^3+^ (LuAG: Ce) @ Sapphire composite.

**
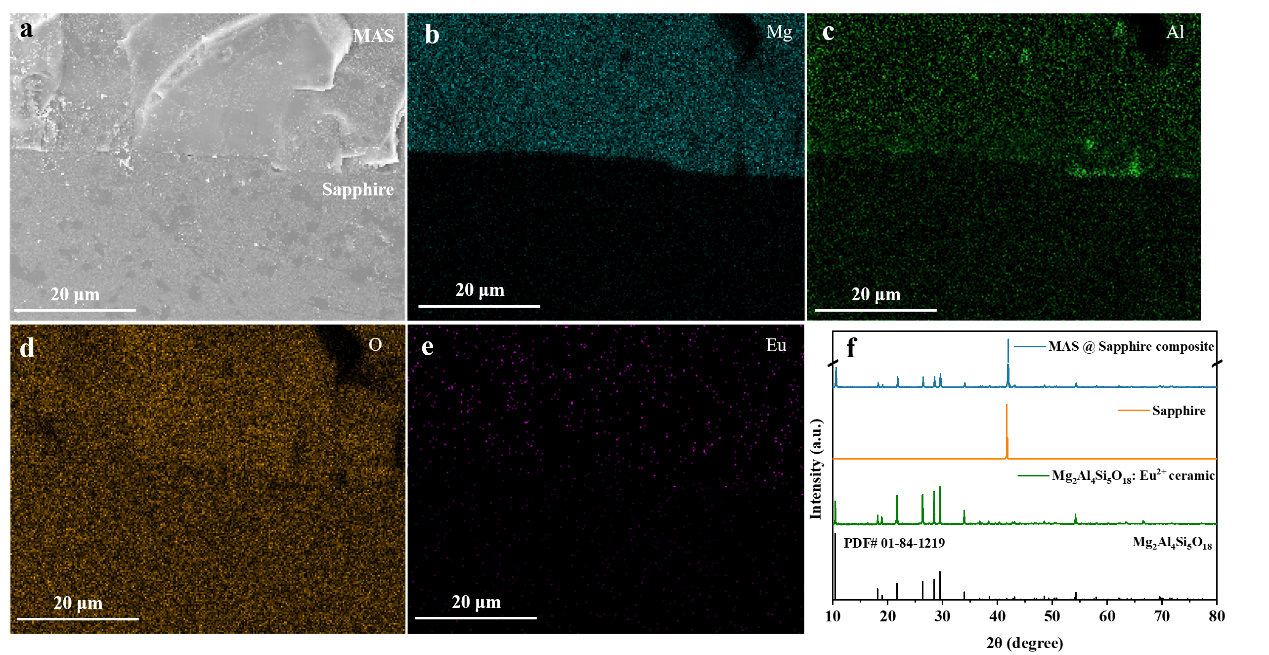
**

**Figure S16.** a) SEM, b-e) EDS mapping and f) XRD pattern of Mg_2_Al_4_Si_5_O_18_: Eu^2+^ (MAS: Eu) @ Sapphire composite.


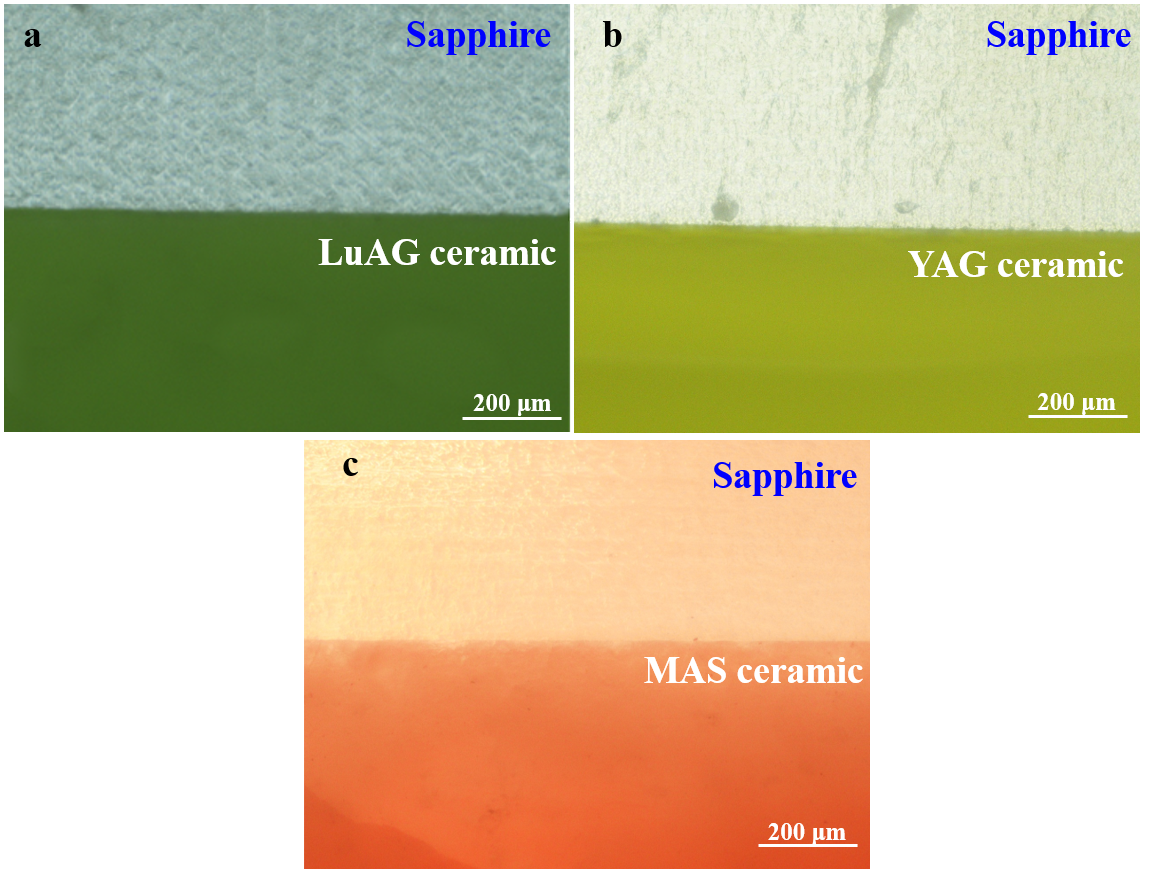


**Figure S17.** Fluorescence images of the a) LuAG: Ce @ Sapphire, b) YAG: Ce @ Sapphire and c) MAS: Eu @ Sapphire composites under 365 nm light excitation.


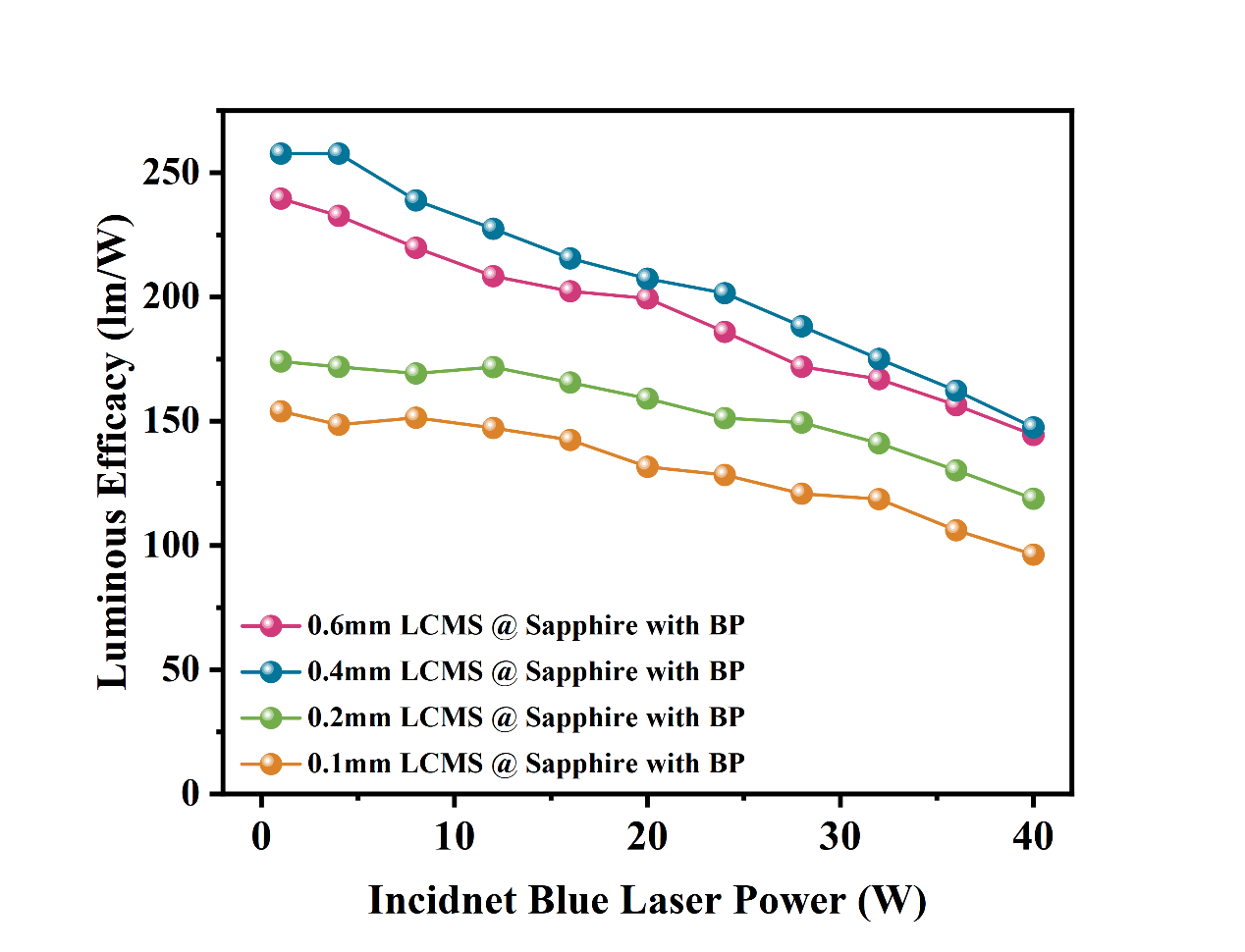


**Figure S18.** LE of LCMS: Ce @sapphire composites with different phosphor ceramic layer thicknesses.

**Figure S19.** Measuring angle dependent color temperature for LCMS: Ce @ sapphire composite.

**Figure S20.** Measuring angle dependent color temperature for commercial color converter.

**
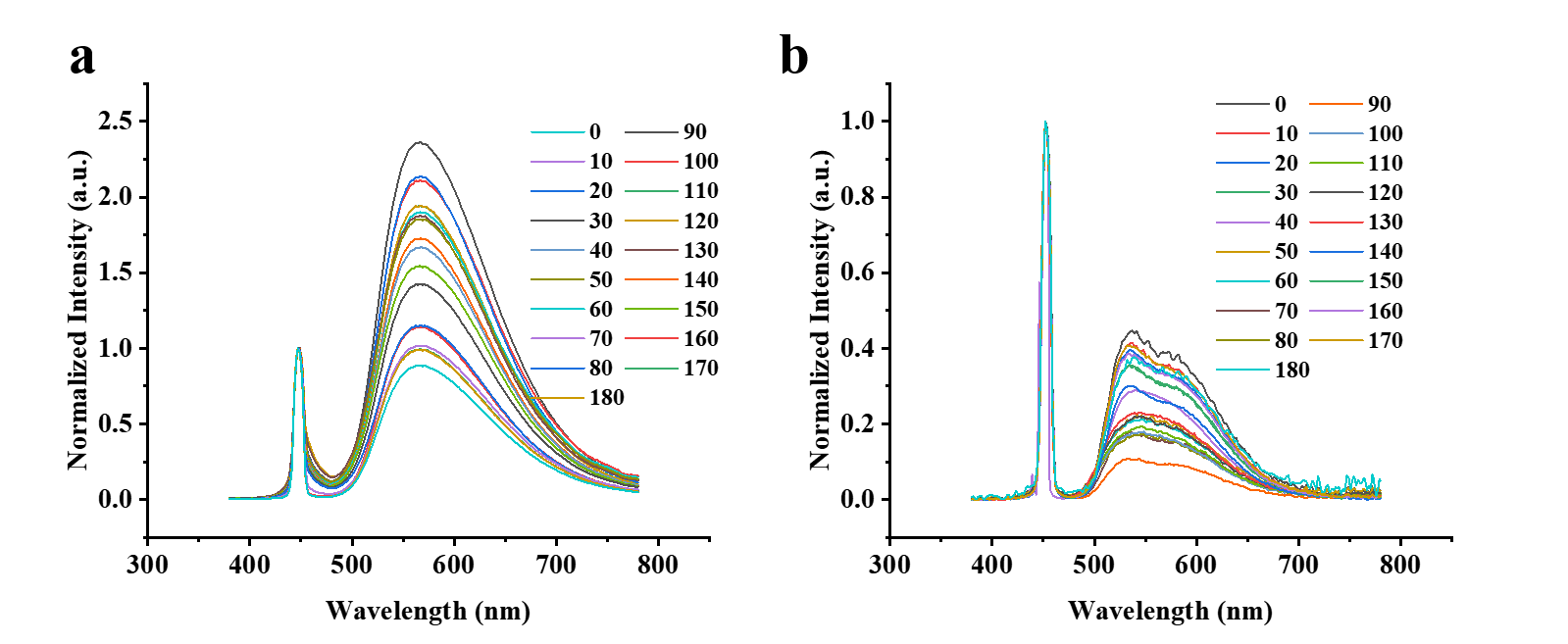
 Figure S21.** Normalized luminescent spectra at different angles (0°-180°) for a) LCMS: Ce @ sapphire composite and b) commercial color converter.


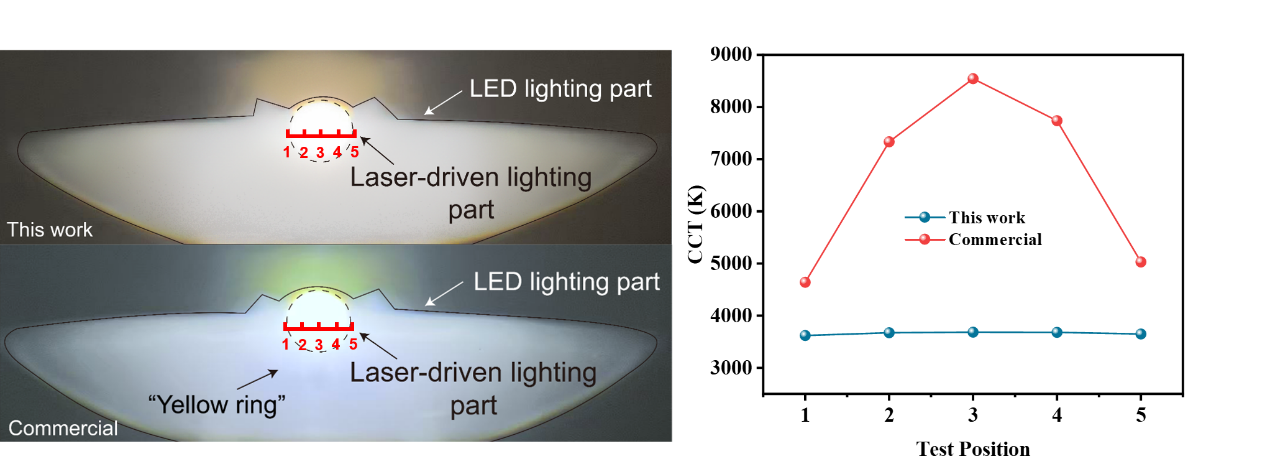


**Figure S22.** The color distribution comparison of LCMS: Ce @ sapphire composite and commercial color converter.


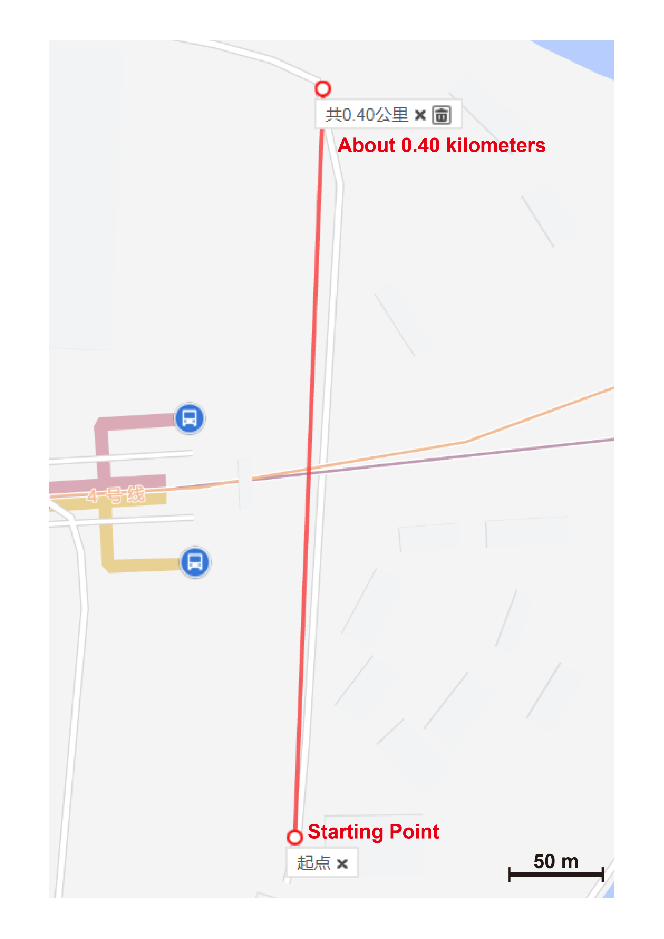


**Figure S23.** The map with clearly marked actual distances for Figure 4e.


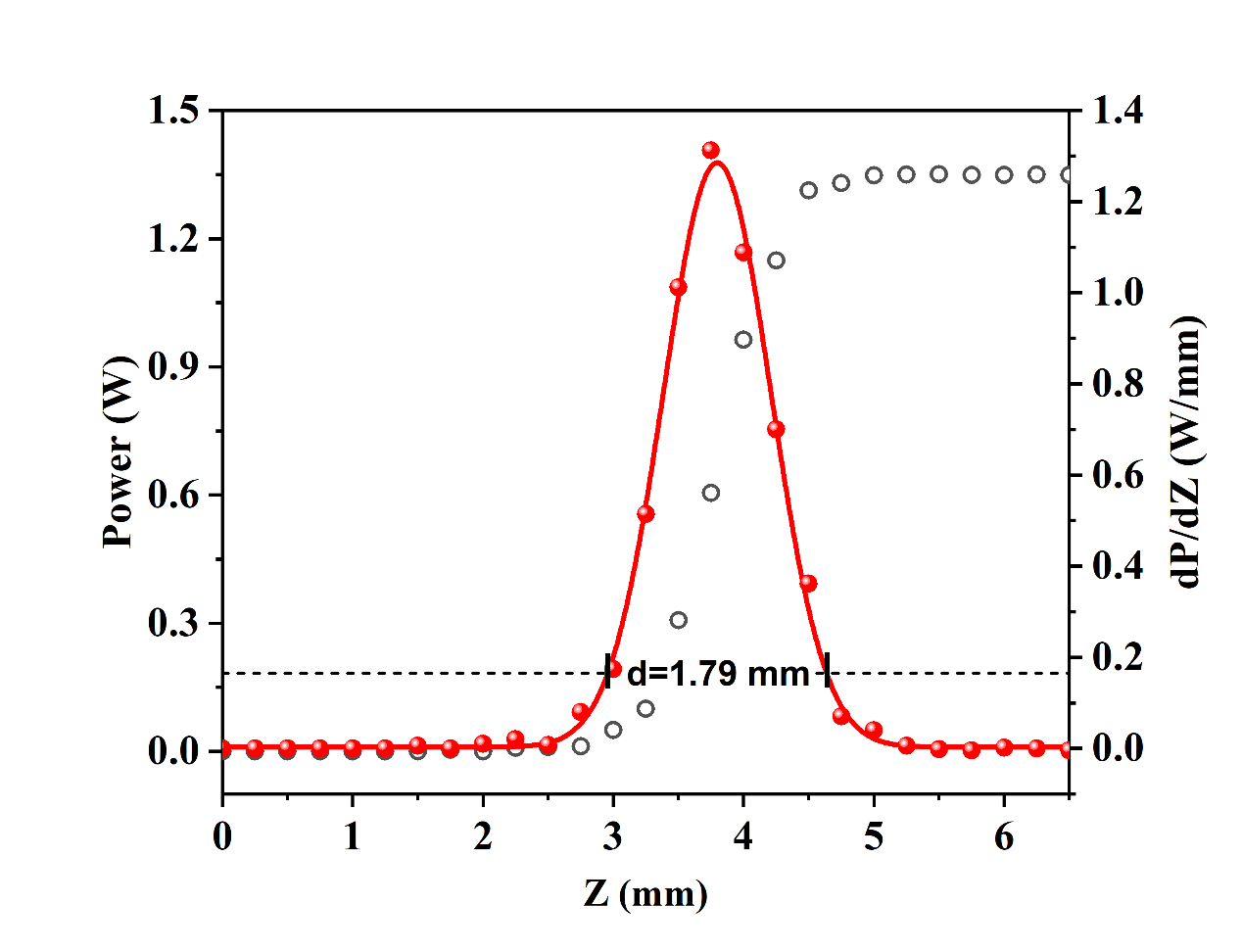


**Figure S24.** The dimensions of the laser spot obtained by the knife-edge method; Left axis records the power intensity as a function of the knife moving length; The first derivative of the power curve (red cycle symbols) is shown in the right axis.

**Discussions on Figure S24**

The laser spot was measured by a knife-edge method by moving a knife-edge across the beam path while monitoring the transmitted power, allowing the spatial intensity distribution and spot size to be extracted via fitting. The diameter d is taken at the point where the intensity falls to 1/e^2^ (approximately 13.5 %) of its maximum, with calculated spot size of 2.54 mm^2^.

**Note S1**

Although the microstructure, which contains porosity, affects optical transparency, this does not preclude its classification as a transparent ceramic. The measured transmittance of Lu_2_CaMg_2_Si_3_O_12_: Ce^3+^ phosphor ceramics can reach 50 %. In fact, these pores can serve as scattering centers, enhancing light extraction efficiency and color uniformity [*Adv. Funct. Mater.*, 2024, 34, 202401026].

**Note S2**

As illustrated in Figure S8, all samples were tested in a transmission mode using a home-built laser-performance measurement platform. To minimize losses from forward emission, a BP (blue-pass) - coated Sapphire was applied to the surface of each sample before testing.

**Note S3**

EL spectra of x = 0.01-0.09 LCMS: Ce @ Sapphire composites were collected under 450 nm blue laser irradiation. P_in_ (the incident blue laser optical power) was fixed at 10000 mW. P_ab_ as the absorbed blue laser optical power, P_re_ (remained blue laser optical power) is the integrated area in the region of 431 nm - 471 nm, while P_em_ (converted optical power) is the integrated area in the region of 472 nm - 750 nm.

**Note S4**

At the saturated P_in_ of 4.75 W/mm^2^, the relative CE drop is 18.8 %, the intensity quenching induced CE drop at this P_in_ is 3.1 %, and so, the thermal quenching induced CE drop is 15.7 %, which converts to 83.5 % contribution of thermal saturation and 16.5 % contribution of optical saturation [*Adv. Funct. Mater.*, 2024, 34, 2307761; *J. Mater. Chem. C*, 2023, 11, 1530].

**Note S5**

The relationship between luminous intensity (I) and driven powers (P) can be expressed by the formula [Adv. Opt. Mater., 2024, 12, 2401672; J. Eur. Ceram. Soc., 2024, 44, 1143]:

$$I \propto P^{n}$$

where n is the number of photons needed to emit a photon. In order to analyze the relationship between P_ab_ and P_em_, the logarithm is taken to the above equation:

$$lg(I) \propto n lg(P)$$

Substituting P_ab_ and P_em_ into the above equation, n can be determined by the slope. In order to avoid the effect of thermal saturation, samples are tested in rotatory mode. As shown in Figure 3e, P_ab_ shows an obvious linear relationship with P_in_ and the value n can be approximately equal to 1 in general, indicating that the absorption is a single-photon process.

After analysis, the best fit should be divided to two stages fitting for P_em_ after removing the saturation points, n_1_ is the slope of P_em1_ when P_in_ ≤ 18 W, n_2_ is the slope of P_em2_ when P_in_ ≥ 20 W. As results, on the one hand, the estimated n_1_ = 0.97 indicates that emitting one photon requires one photon, on the other hand, the estimated n_2_ = 1.05 indicates that emitting one photon requires more than one photon. Therefore, it can be presumed the optical intensity saturation is composed of the single-photon and the double-photon processes.
